# Supplementary material for: Transcriptomic responses to endurance exercise training in rats
Source: BMC Genom Data. 2026 Jun 21;27:74. doi: 10.1186/s12863-026-01440-8 (PMC13548577; doi:10.1186/s12863-026-01440-8)
Supplement: Supplementary file 1 [file 12863_2026_1440_MOESM1_ESM.pdf]

Transcriptomic responses to endurance exercise training in rats: Supplemental File

Conrad Oakes[1] **and Lior Pachter**[1,2]

[1]Division of Biology and Biological Engineering, California Institute of Technology, Pasadena, CA, 91125, USA
[2]Department of Computing and Mathematical Sciences, California Institute of Technology, Pasadena, CA, 91125, USA

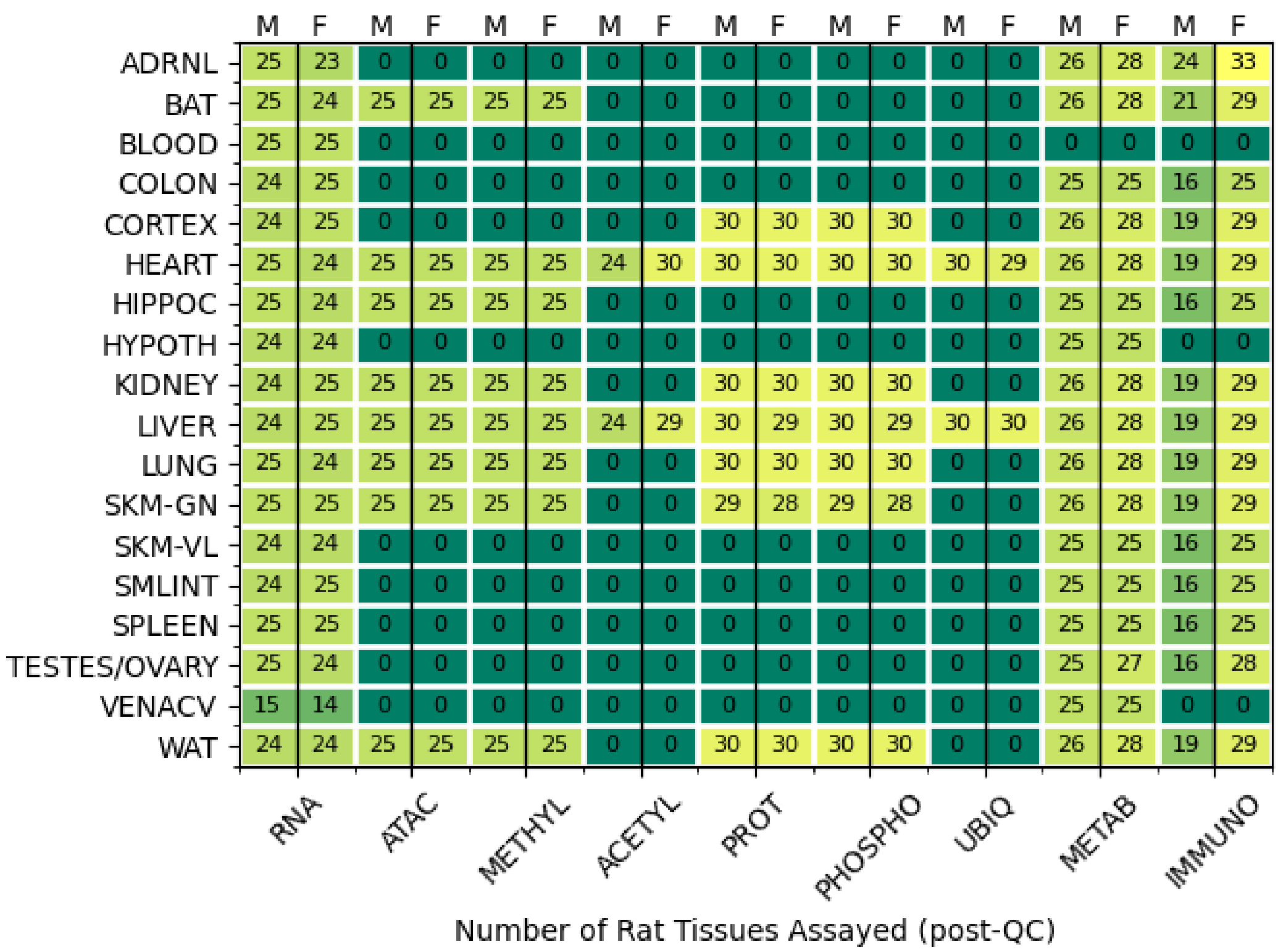

Fig. 1. Samples of Each Tissue Available for Each Assay post-QC. An overview of number of samples were collected and passed quality control for each assay in each tissue, split by sex.

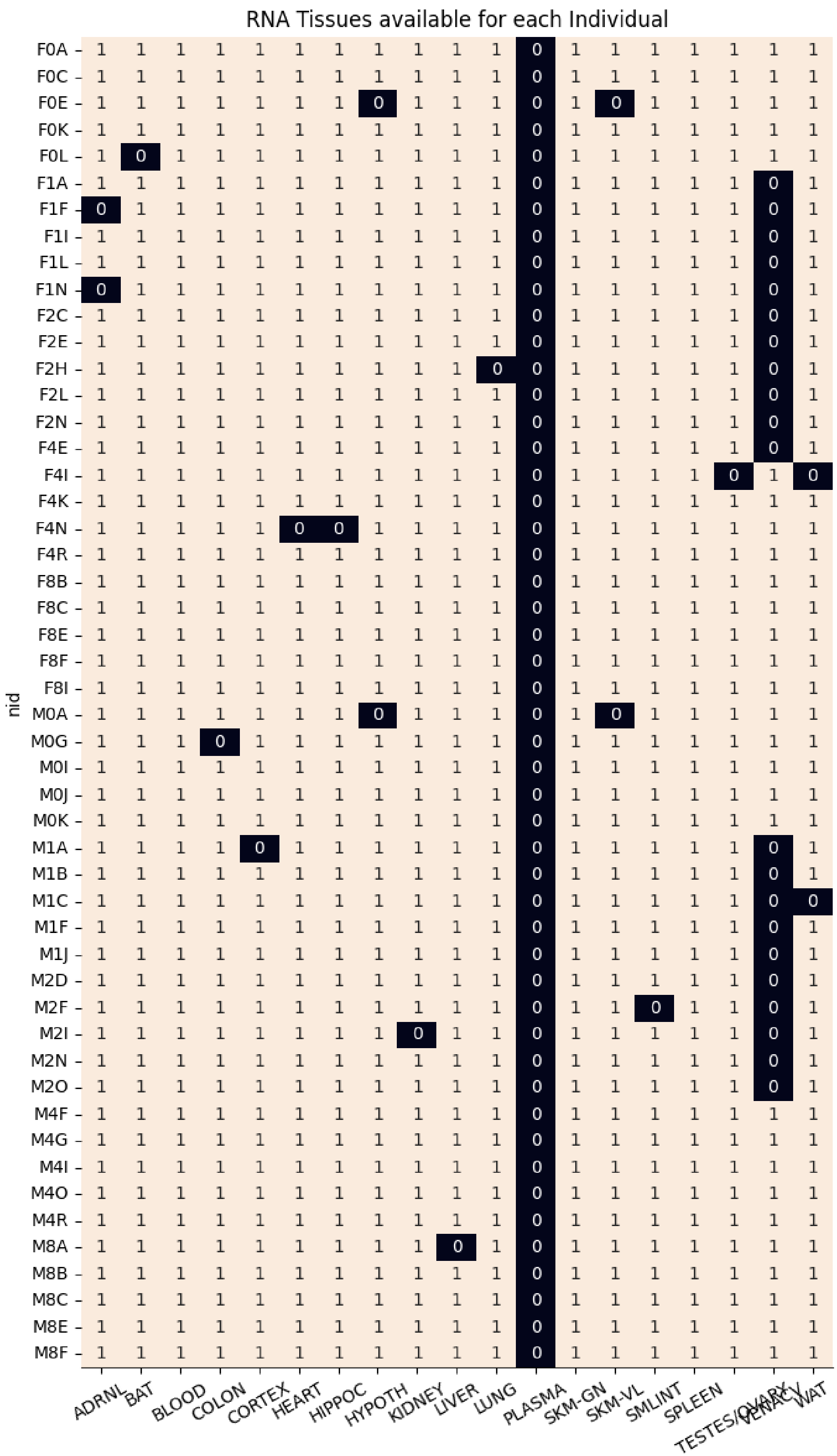

Fig. 2. RNA Tissues Available for Each Individual. A binary heatmap showing which RNA sequencing tissues were successfully collected and passed quality control for each individual rat (rows), across all tissue types (columns).

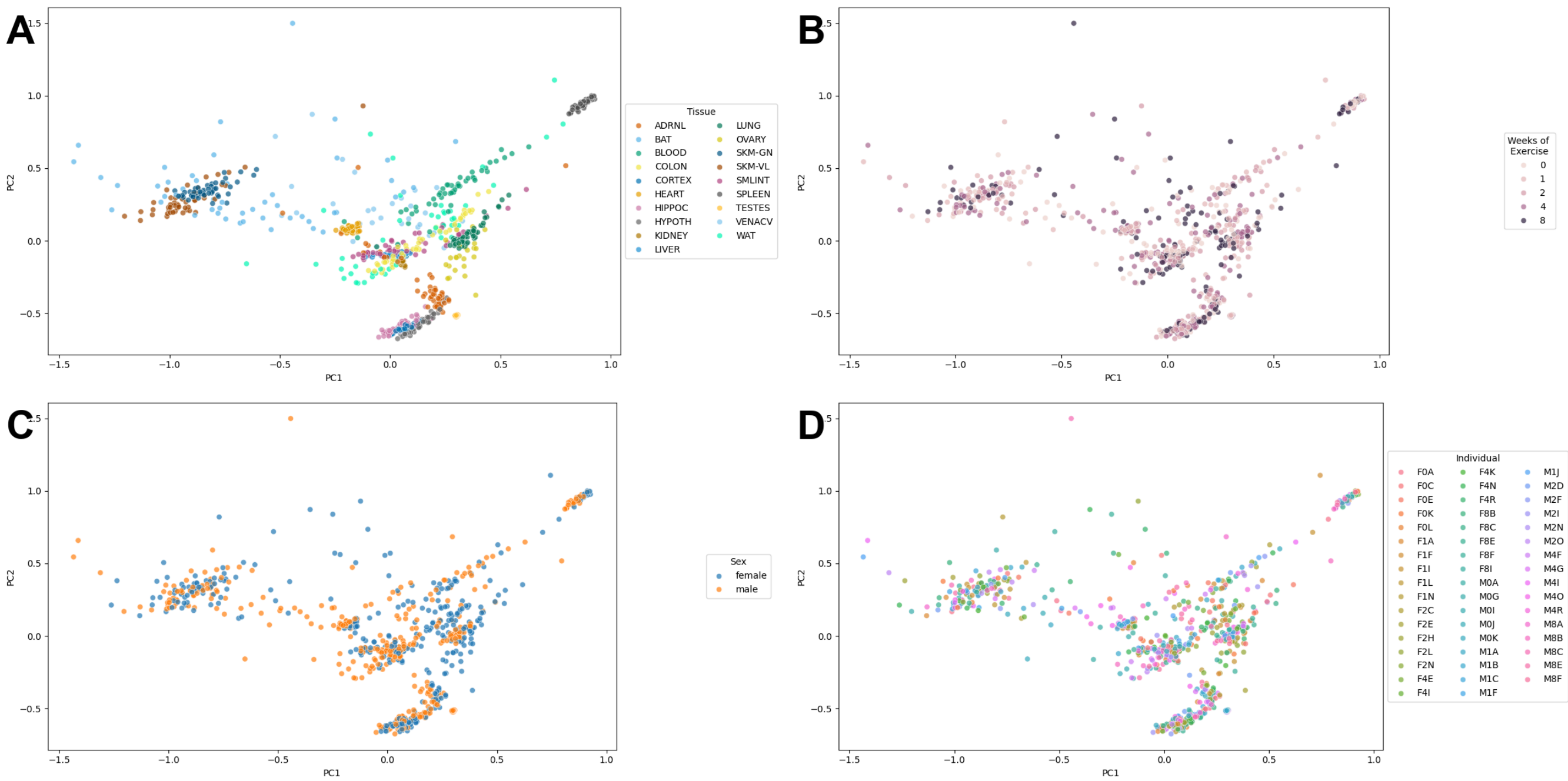

Fig. 3. PCAs of data post-scVI batch correction. PCA plots of the data post-scVI batch correction for tissue and sex differences. Plots highlighted by A) Tissue, B) Time, C) Sex, and D) Individual.

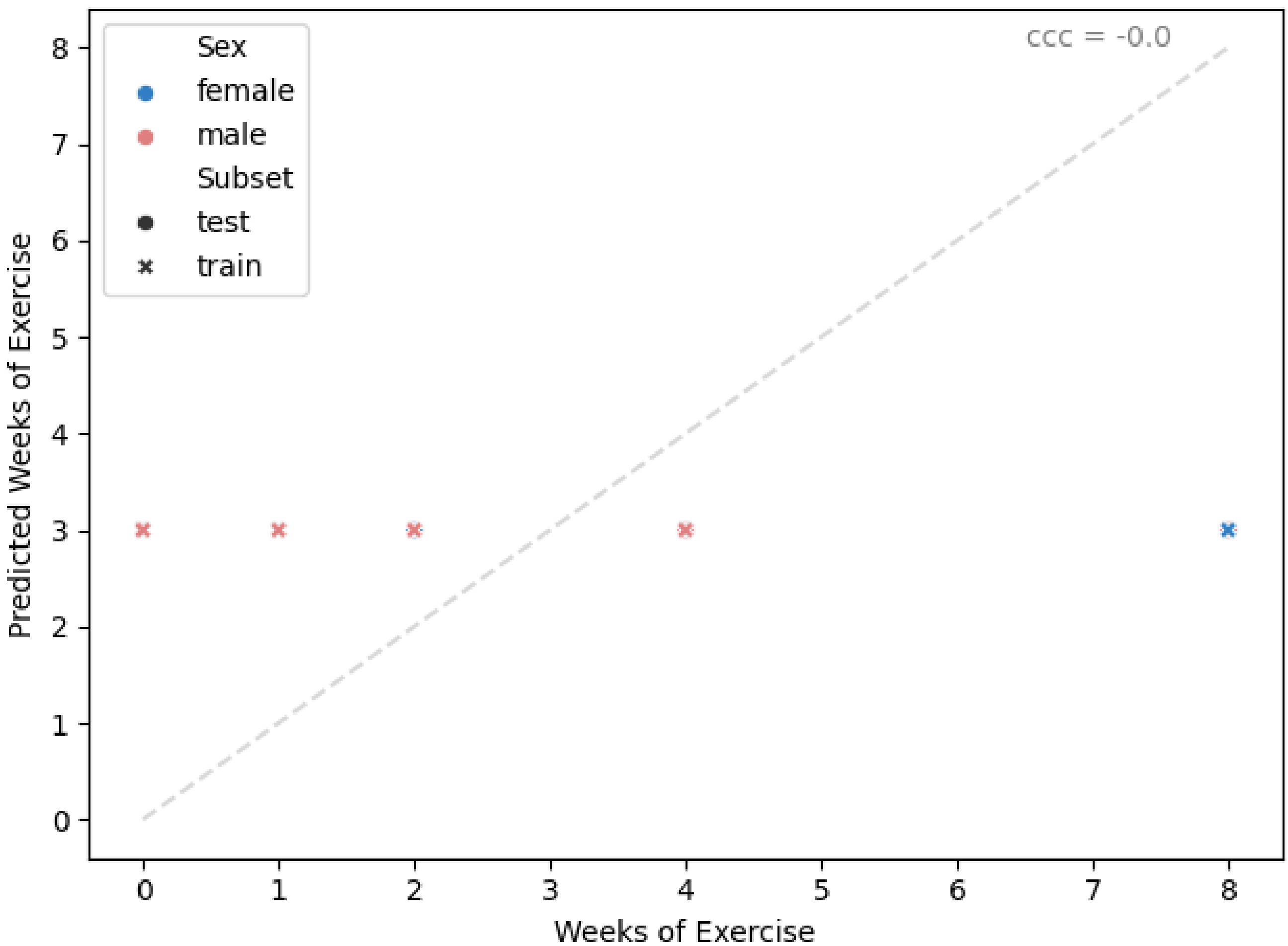

Fig. 4. Linear Regression to Predict Weeks of Exercise using scVI Latent Representation. The results of generating a Generalized Linear Model to predict weeks of exercise using the scVI latent space representation of the RNA data across all tissues.

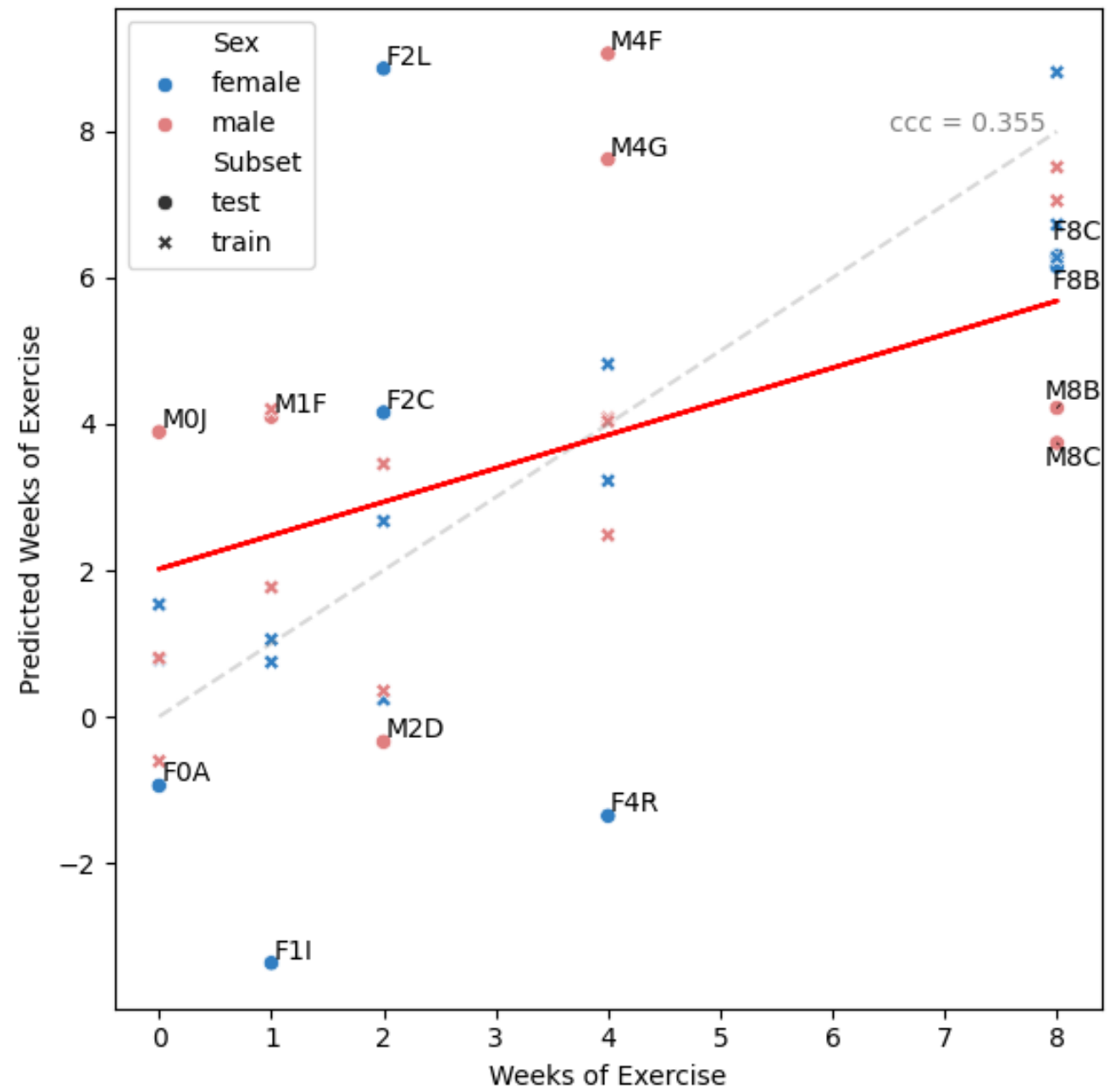

Fig. 5. Linear Regression to Predict Weeks of Exercise using UMI data from each tissue combined. Predicted weeks of exercise (y-axis) versus actual weeks of exercise (x-axis), colored by sex and shaped by train/test subset.

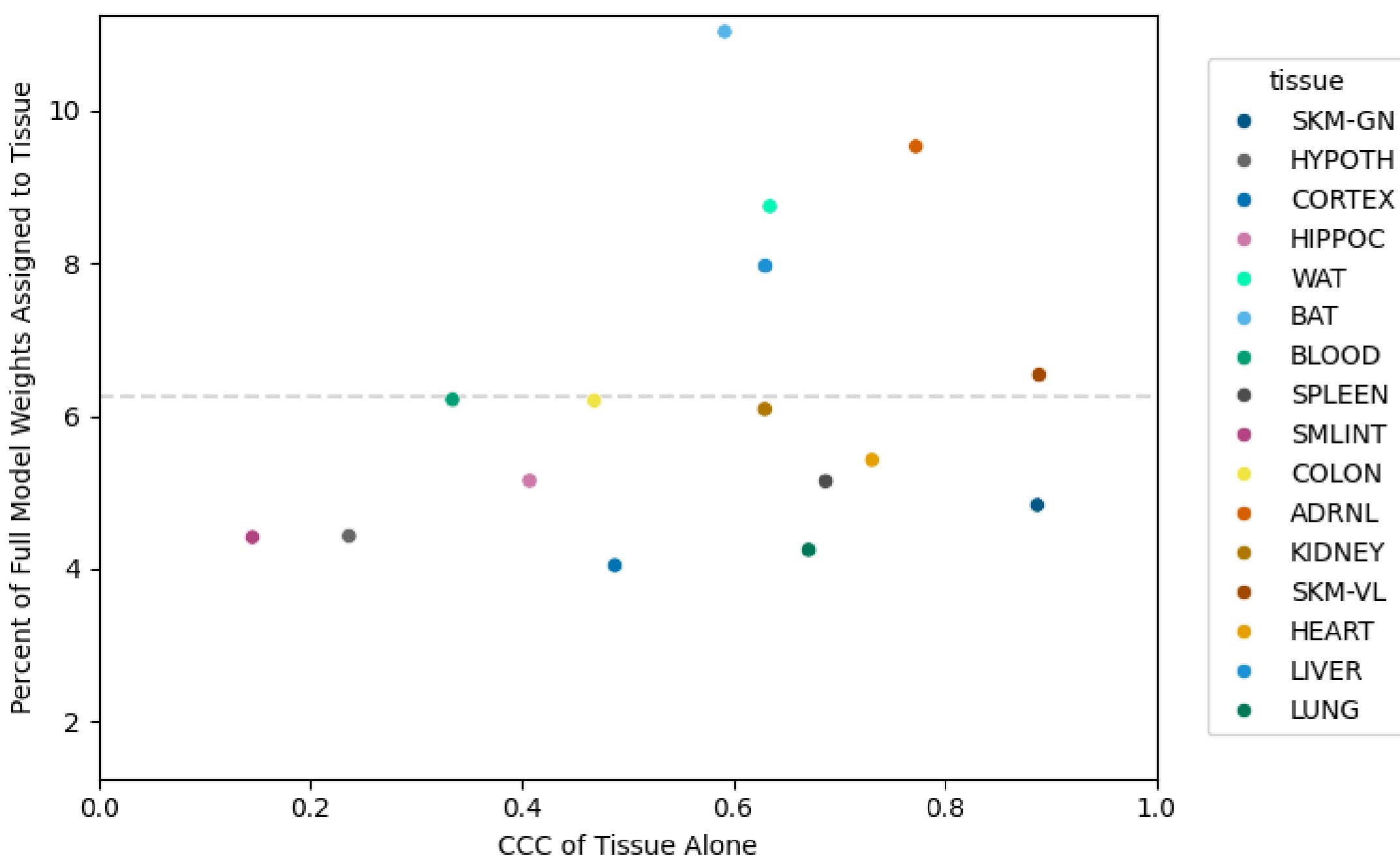

Fig. 6. Comparison of individual tissue Linear Regressions to overall, combined linear regression. The x-axis is the concordance correlation coefficient of each tissue when used to predict weeks of exercise with a GLM. The y-axis is the percentage of full model weights that were assigned to that tissue, with the dotted line indicating the value one would expect if each tissue contributed an equal amount of weight to the full model.

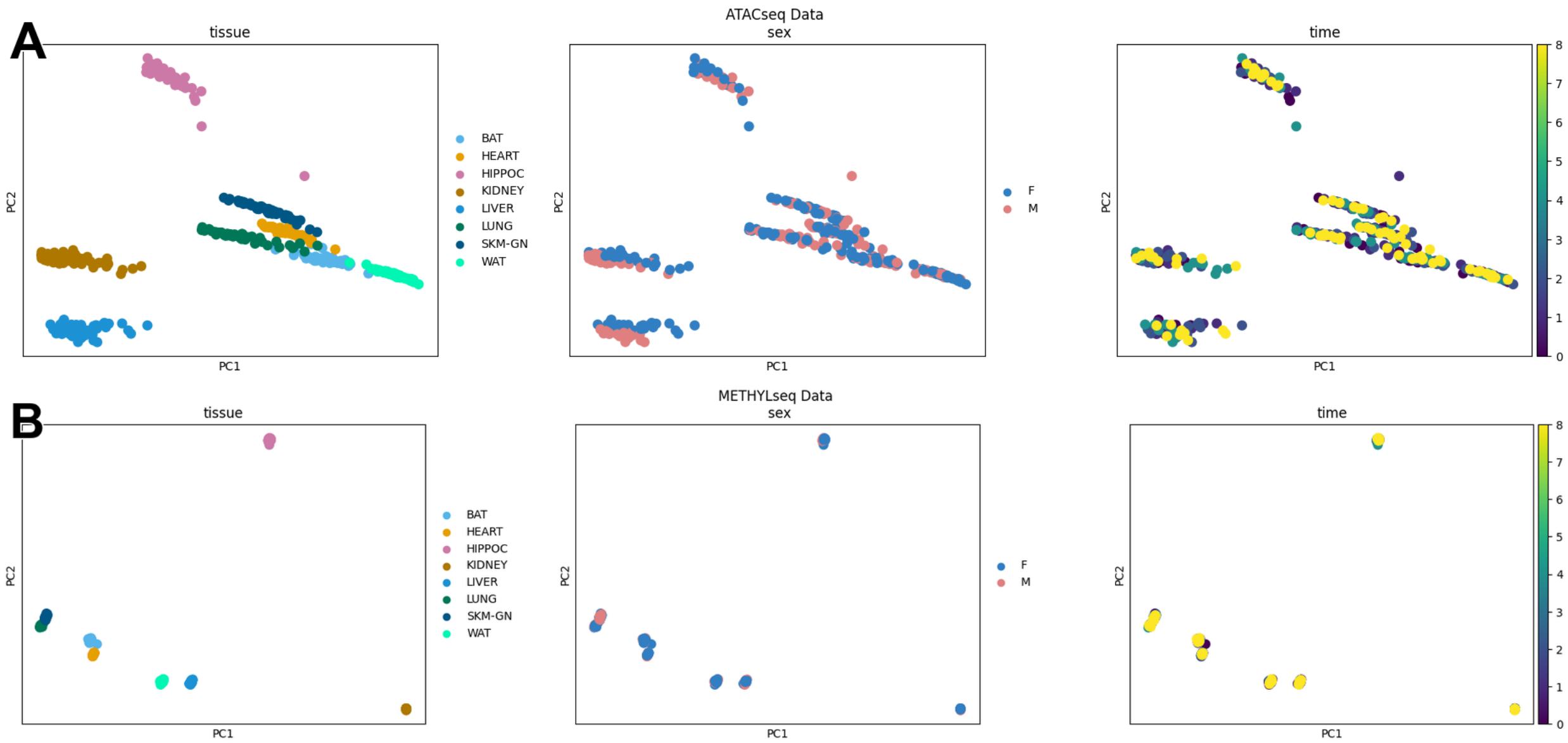

Fig. 7. PCA of ATACseq and METHYLseq data. PCA plots of A) ATACseq data and B) METHYLseq data, highlighted by tissue, sex, and time.

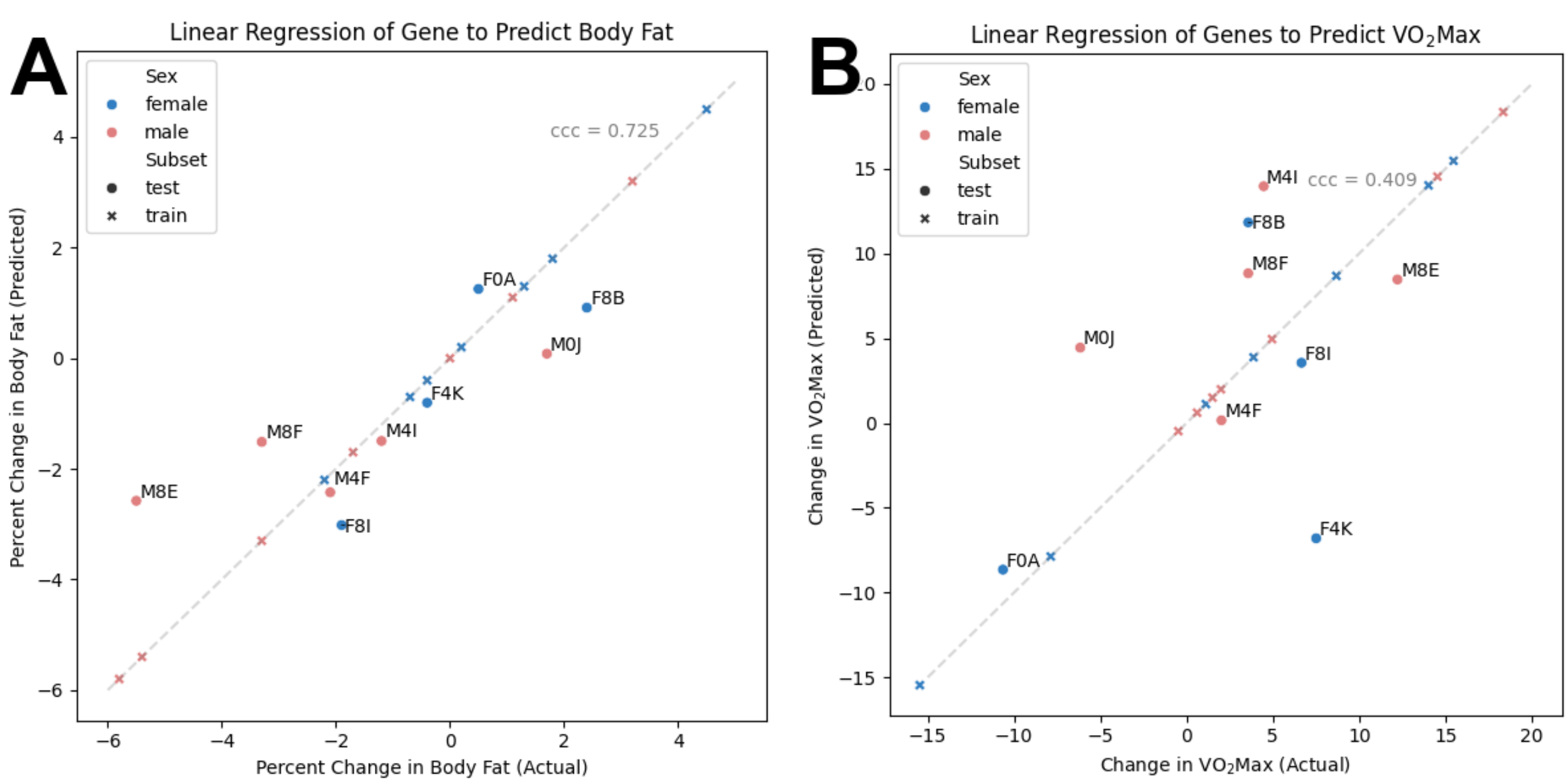

Fig. 8. Linear Regression to Predict Physiological Measurements. The results of generating a Generalized Linear Model to predict A) Percent Change in Body Fat and B) Change in VO_2 Max.

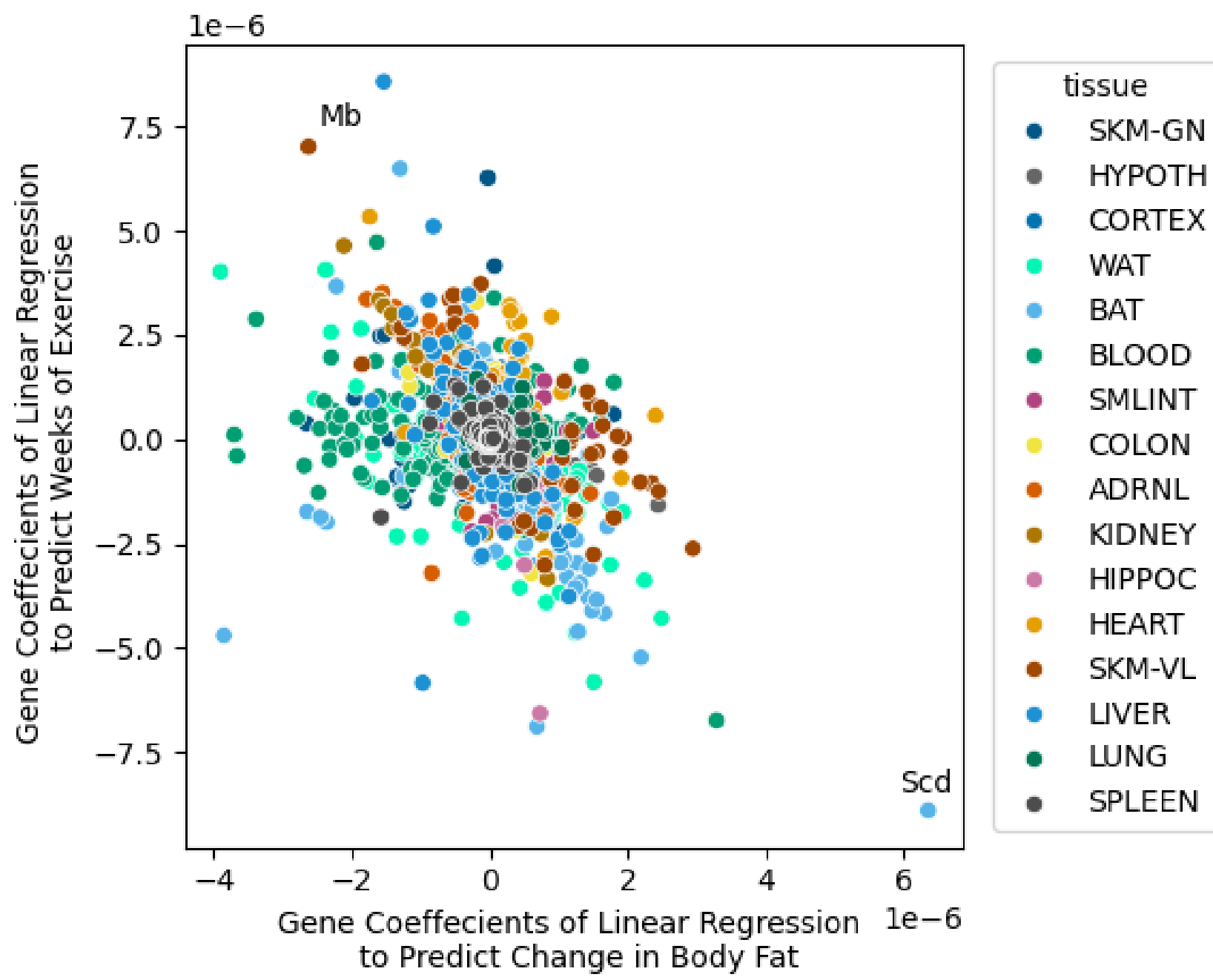

Fig. 9. Linear Regression Model Weights for Genes when predicting Percent Change in Body Fat vs when predicting Weeks of Exercise. The model weight coefficients for each gene when used to predict weeks of exercise (y-axis) or percent change in body fat (x-axis), colored by tissue.

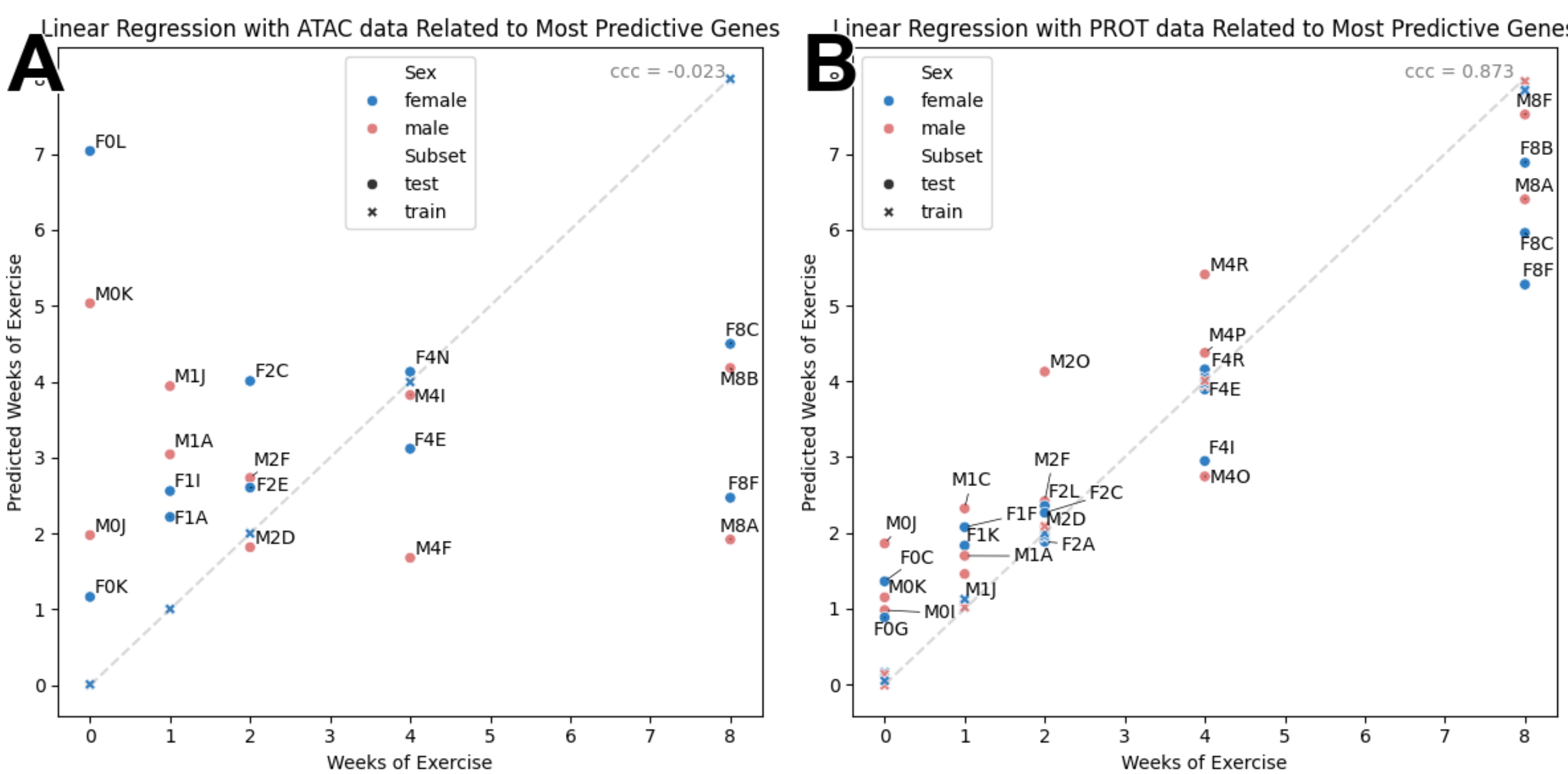

Fig. 10. Linear regression to predict weeks of exercise for A) ATAC data and B) PROT data after filtering for the genes most predictive in the RNA linear regression.

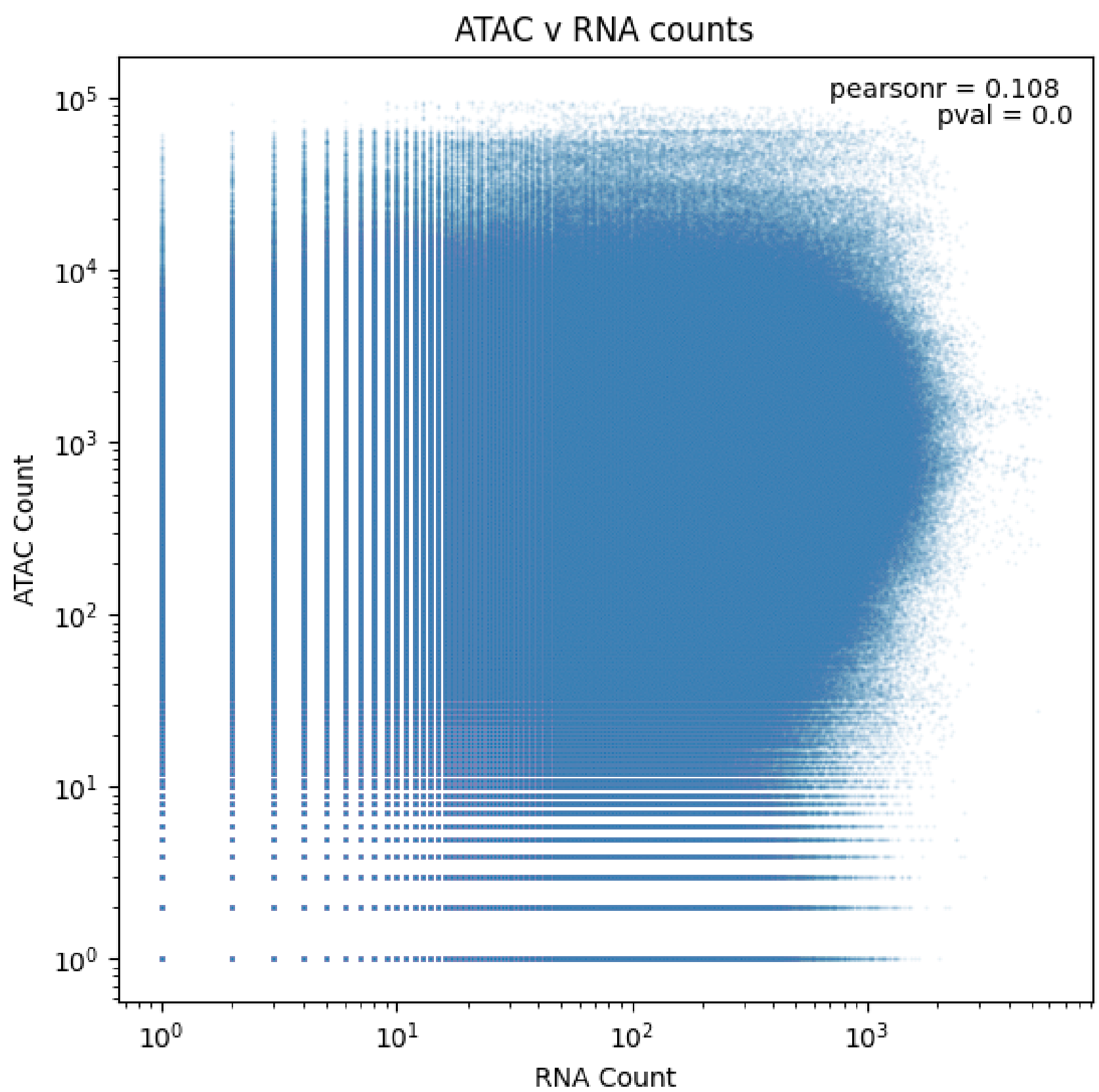

Fig. 11. For each gene, correlation between its RNA counts and ATAC counts for its related promoters within 1 kb.

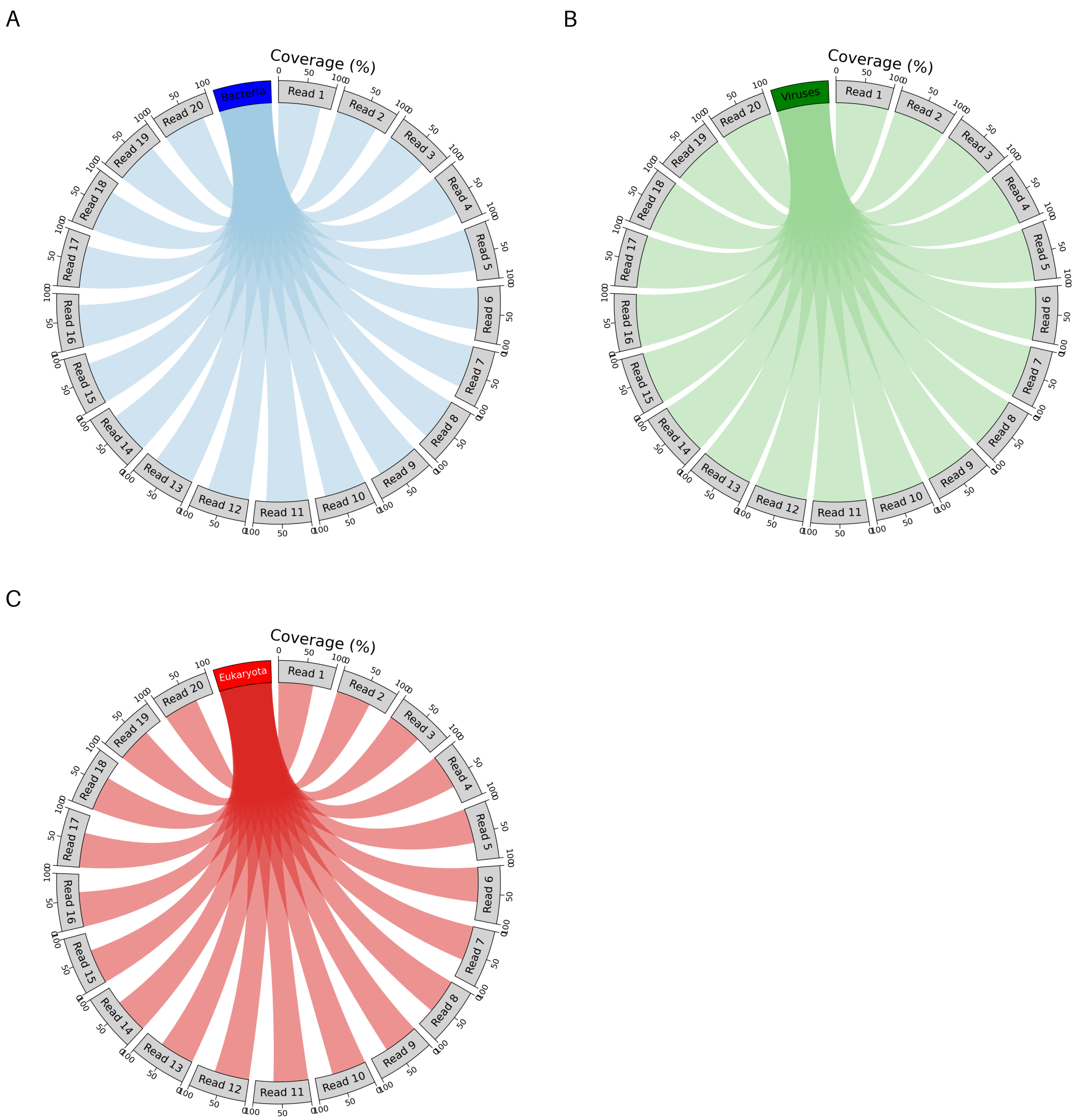

Fig. 12. pyCirclize (https://github.com/moshi4/pyCirclize) plots showing the BLAST results of sequencing reads for putative virus u234187 randomly selected from rat F0K, separated by best-hit superkingdom: A) Bacteria, B) Viruses, and C) Eukaryota. Each outer sector corresponds to one sequencing read, and the connecting links show alignment to the respective superkingdom. The width of each link indicates the BLAST alignment coverage percentage.

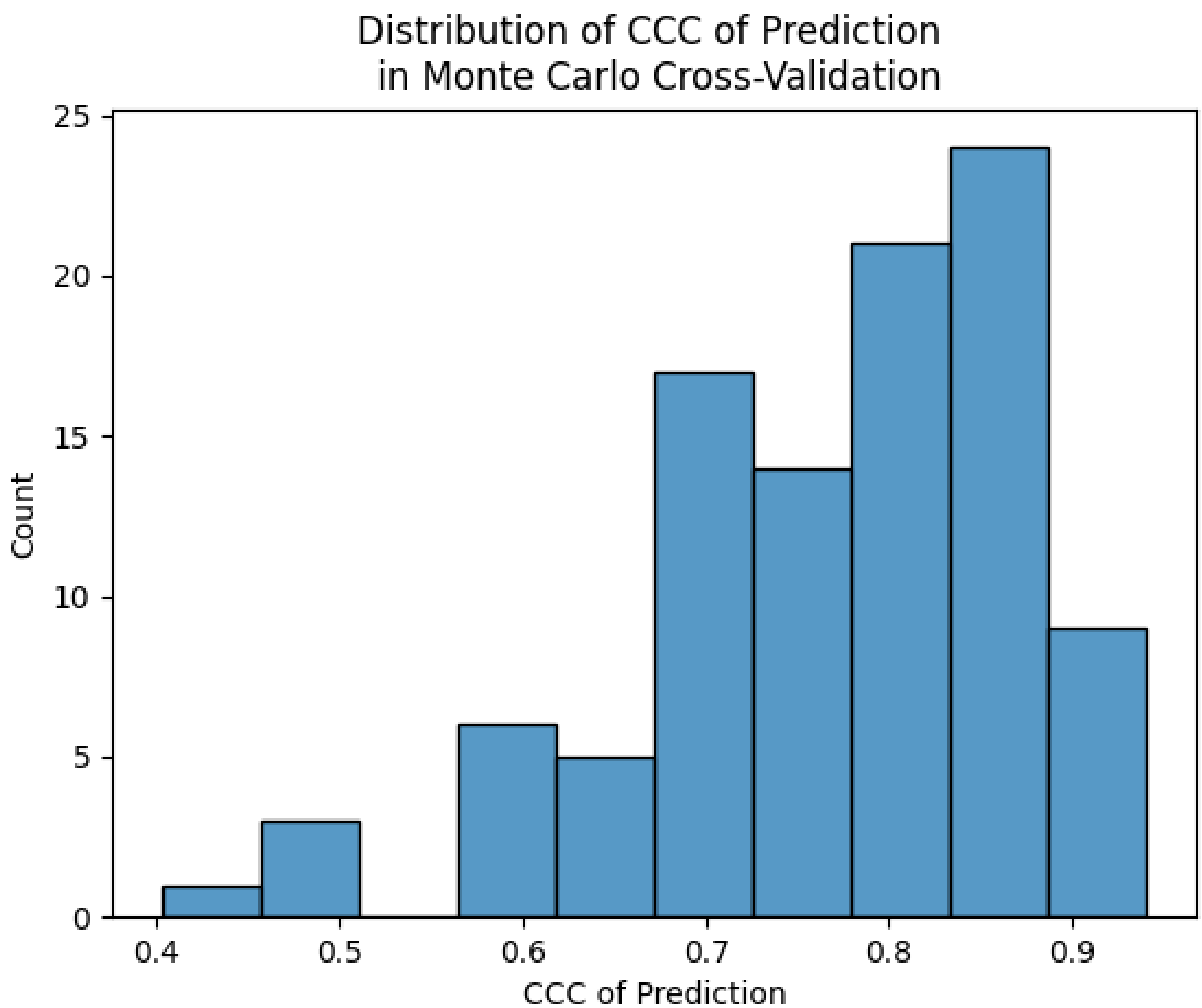

Fig. 13. Distribution of Concordance Correlation Coefficient (CCC) across Monte Carlo Cross-Validation iterations of the full multi-tissue RNA linear regression model predicting weeks of exercise.

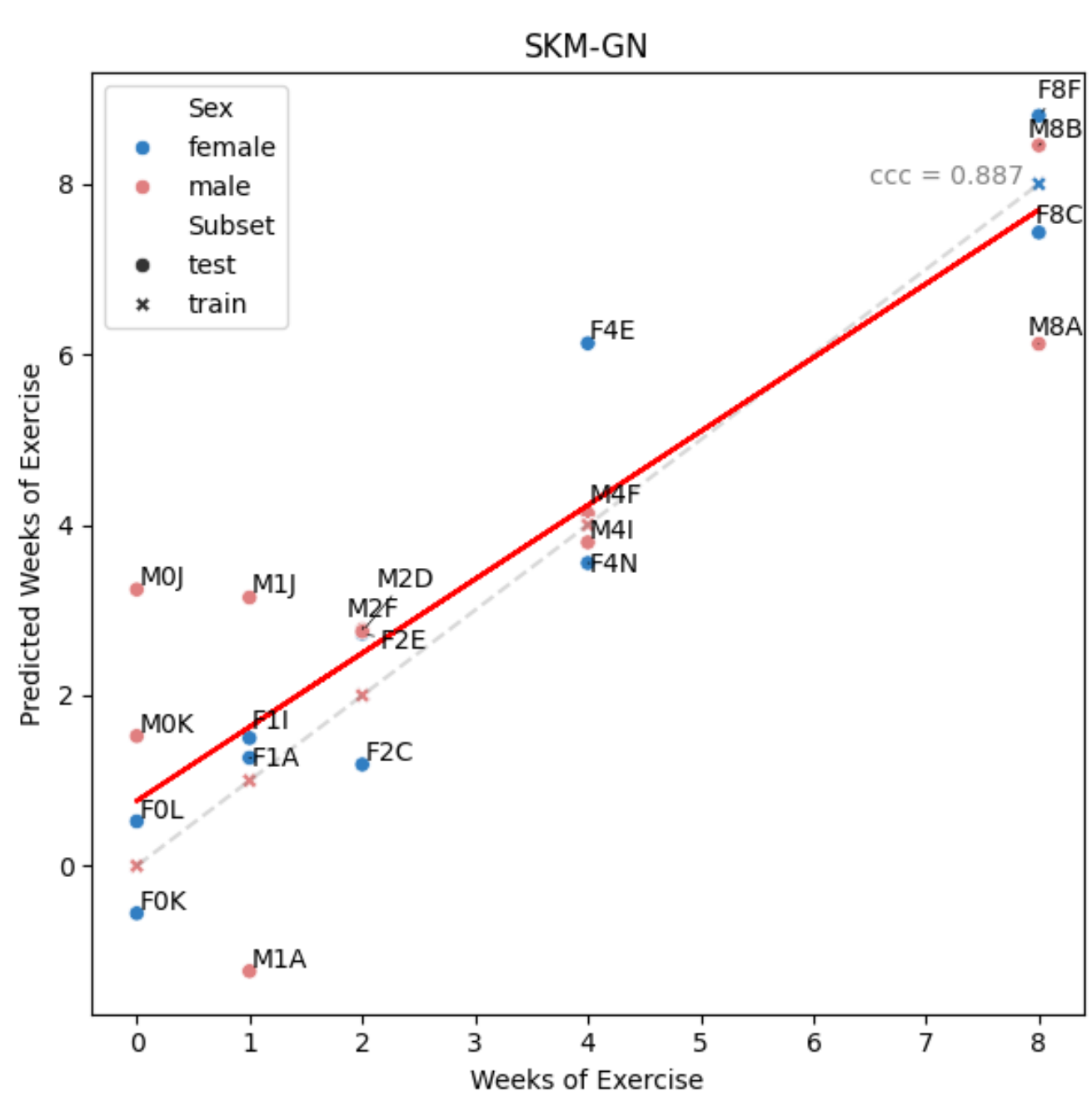

Fig. 14. Linear regression to predict weeks of exercise using RNA data from skeletal muscle gastrocnemius (SKM-GN) alone. Points are colored by sex and shaped by train/test subset. The dashed grey line indicates perfect prediction and the red line is the fit line.

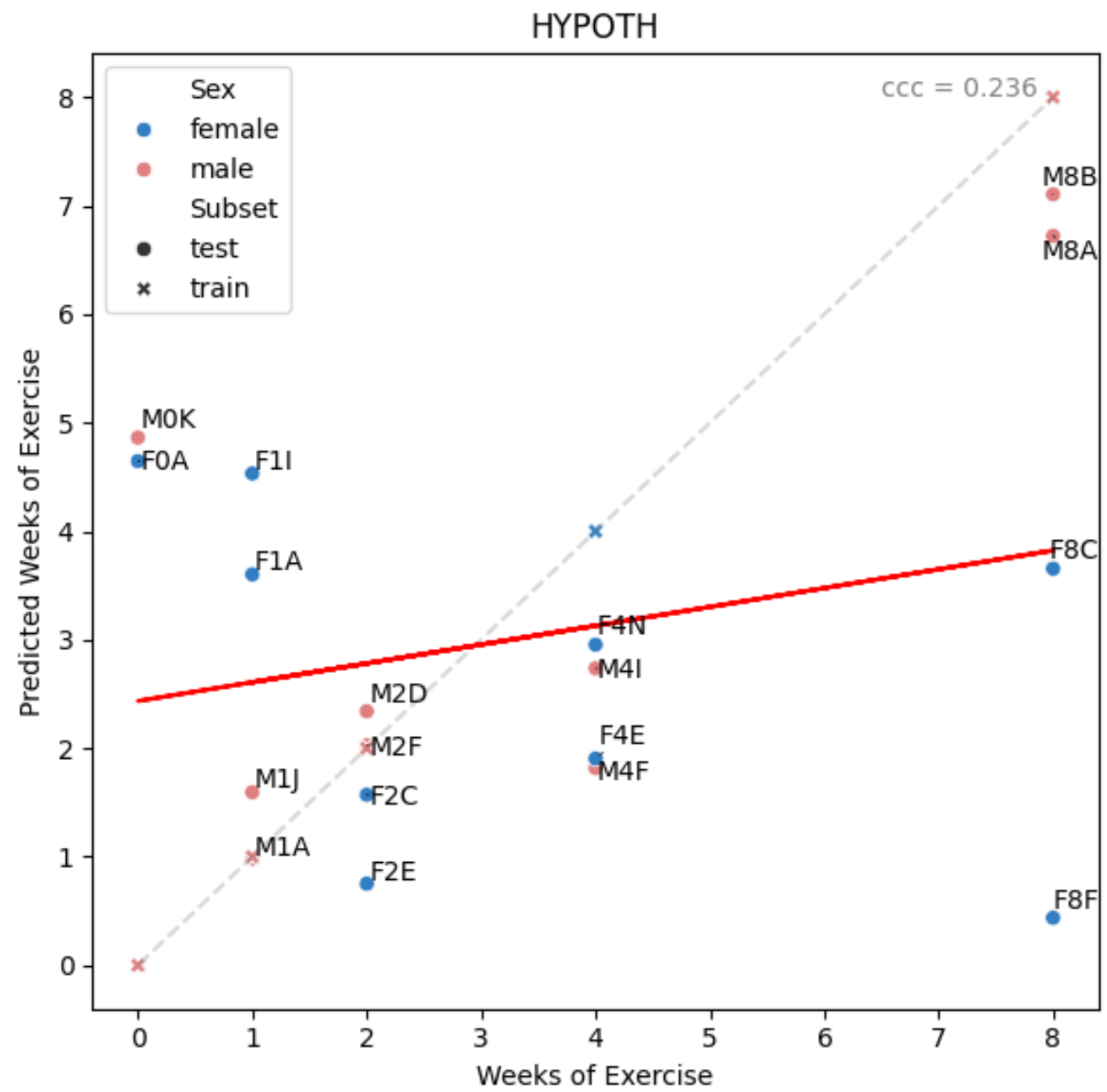

Fig. 15. Linear regression to predict weeks of exercise using RNA data from hypothalamus (HYPOTH) alone. Points are colored by sex and shaped by train/test subset.

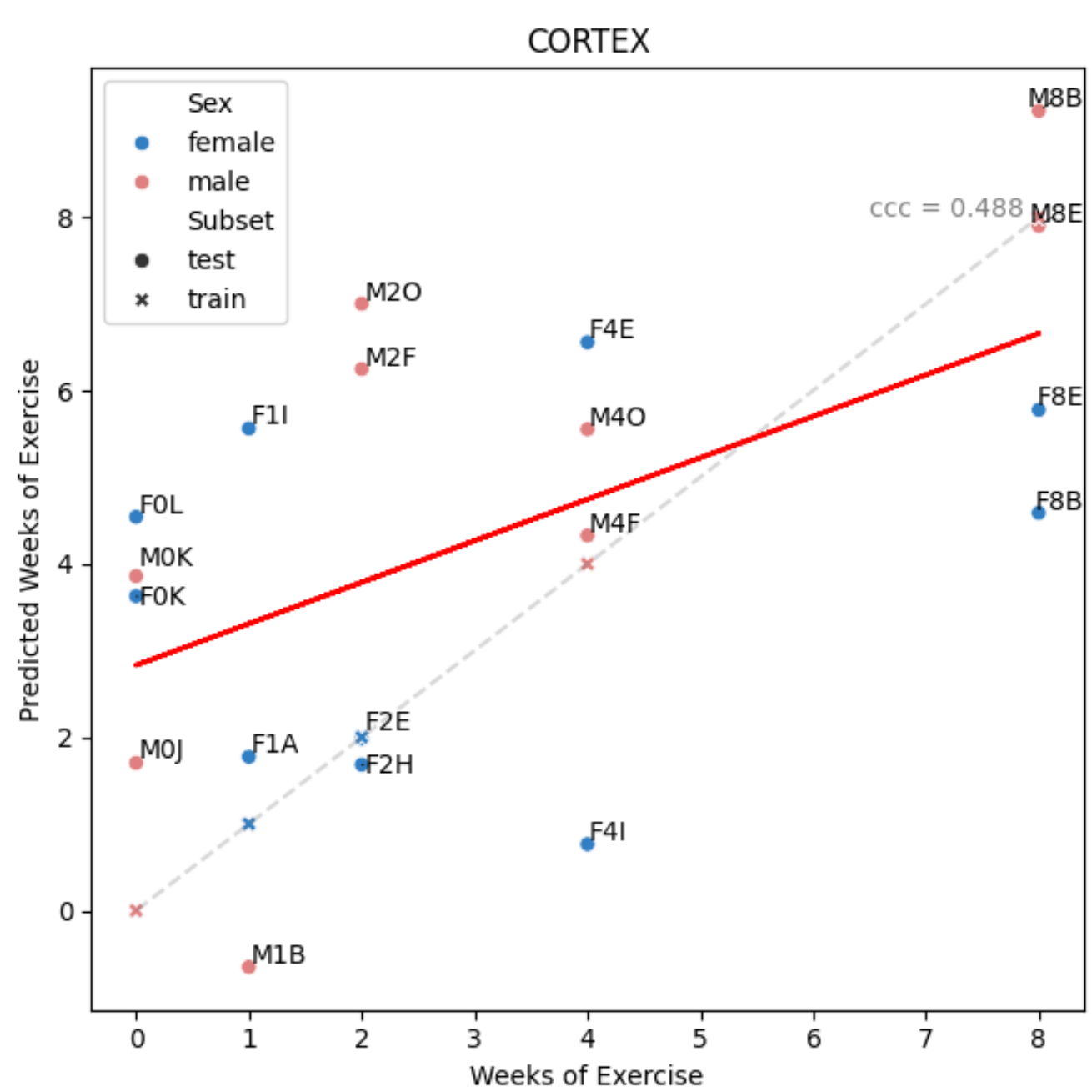

Fig. 16. Linear regression to predict weeks of exercise using RNA data from cortex (CORTEX) alone. Points are colored by sex and shaped by train/test subset.

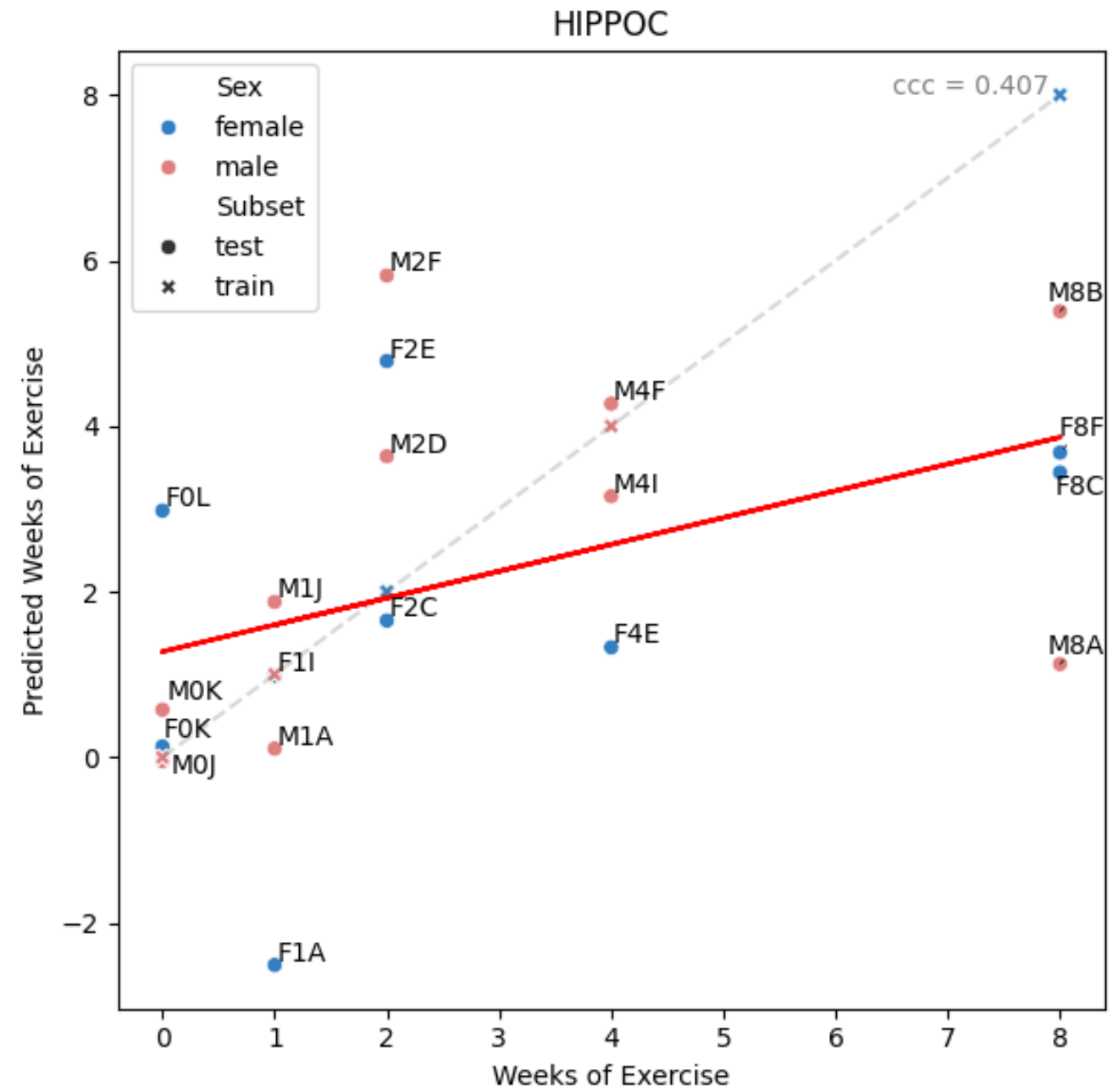

Fig. 17. Linear regression to predict weeks of exercise using RNA data from hippocampus (HIPPOC) alone. Points are colored by sex and shaped by train/test subset.

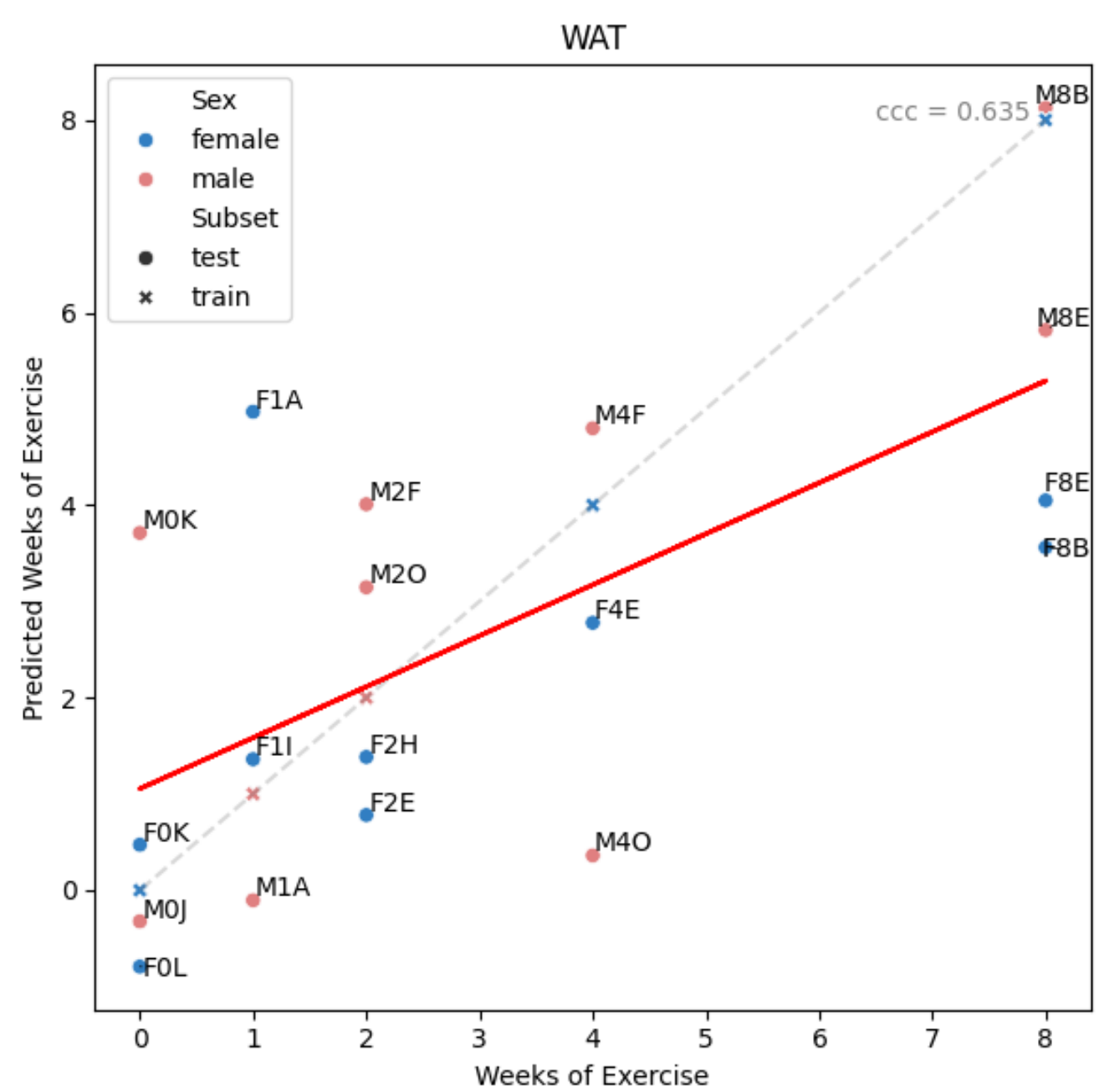

Fig. 18. Linear regression to predict weeks of exercise using RNA data from white adipose tissue (WAT) alone. Points are colored by sex and shaped by train/test subset.

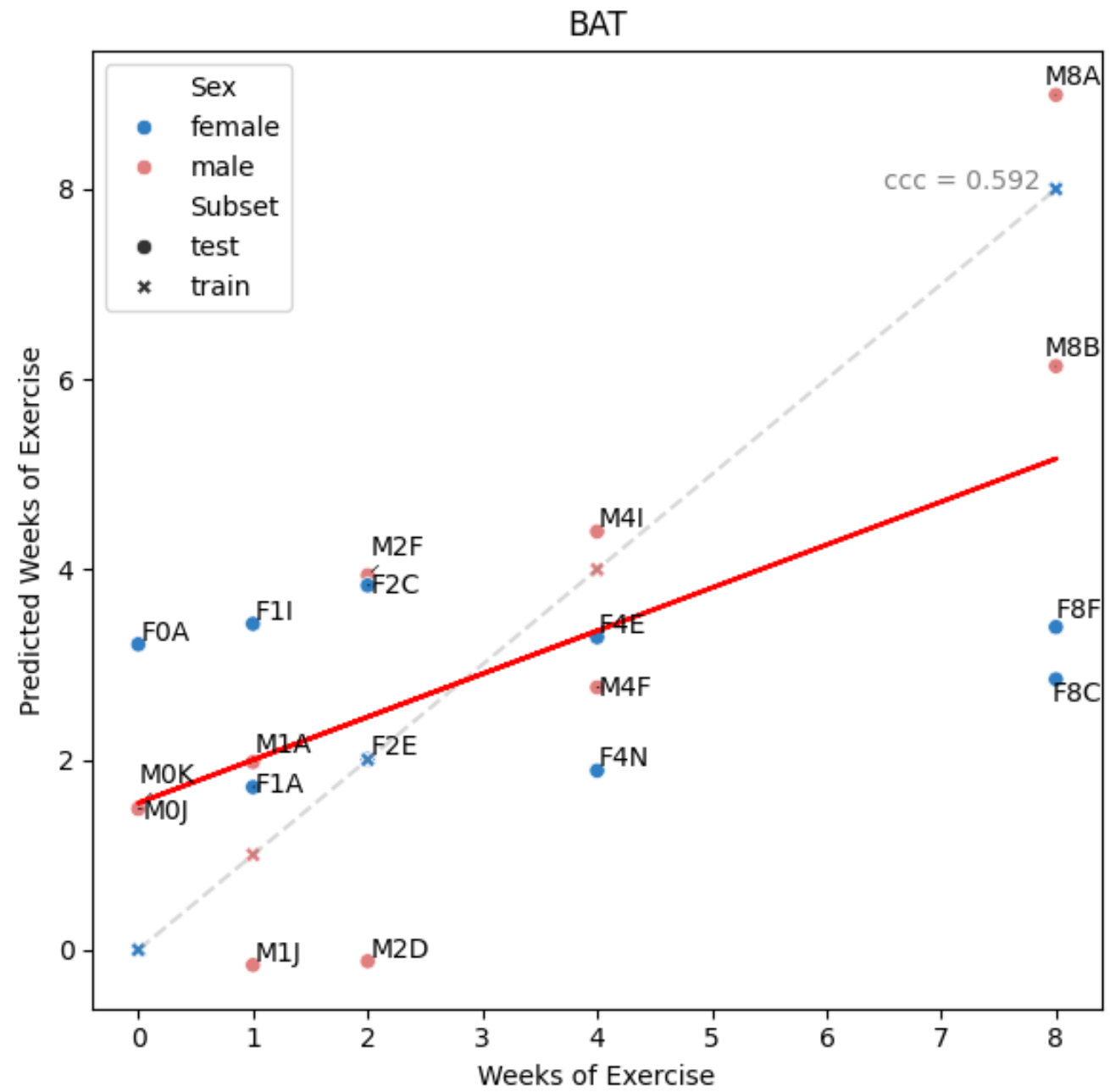

Fig. 19. Linear regression to predict weeks of exercise using RNA data from brown adipose tissue (BAT) alone. Points are colored by sex and shaped by train/test subset.

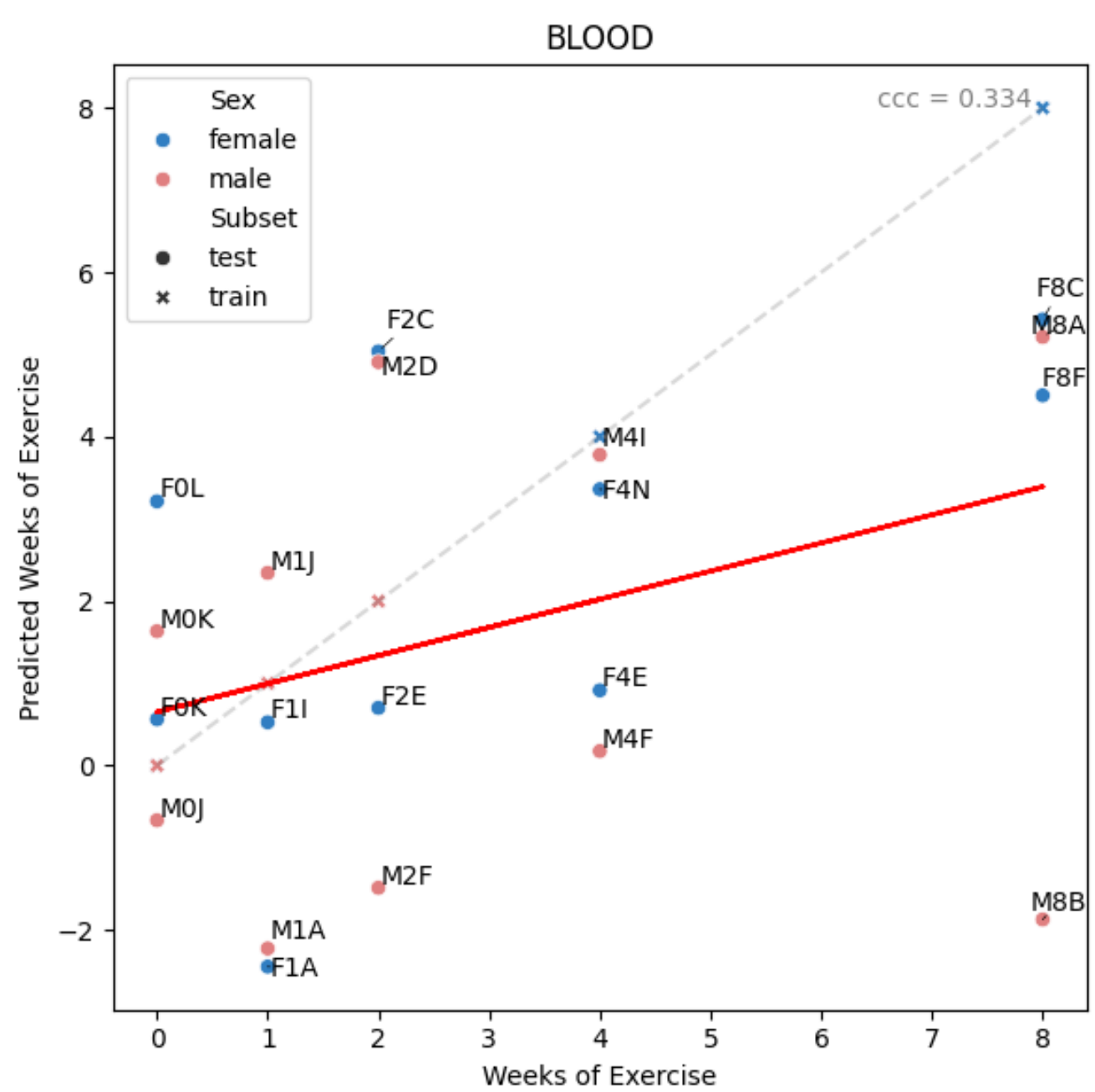

Fig. 20. Linear regression to predict weeks of exercise using RNA data from blood (BLOOD) alone. Points are colored by sex and shaped by train/test subset.

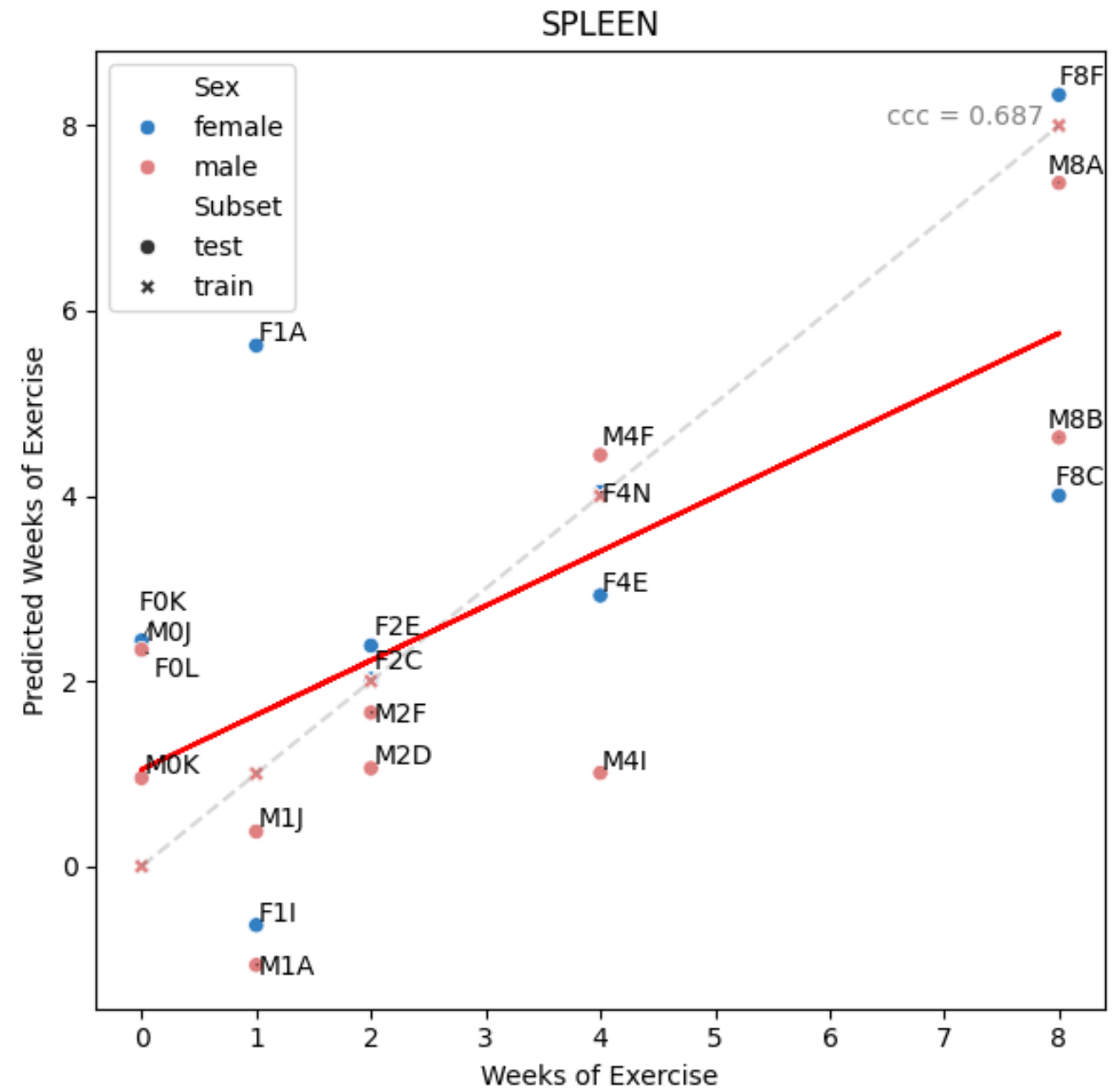

Fig. 21. Linear regression to predict weeks of exercise using RNA data from spleen (SPLEEN) alone. Points are colored by sex and shaped by train/test subset.

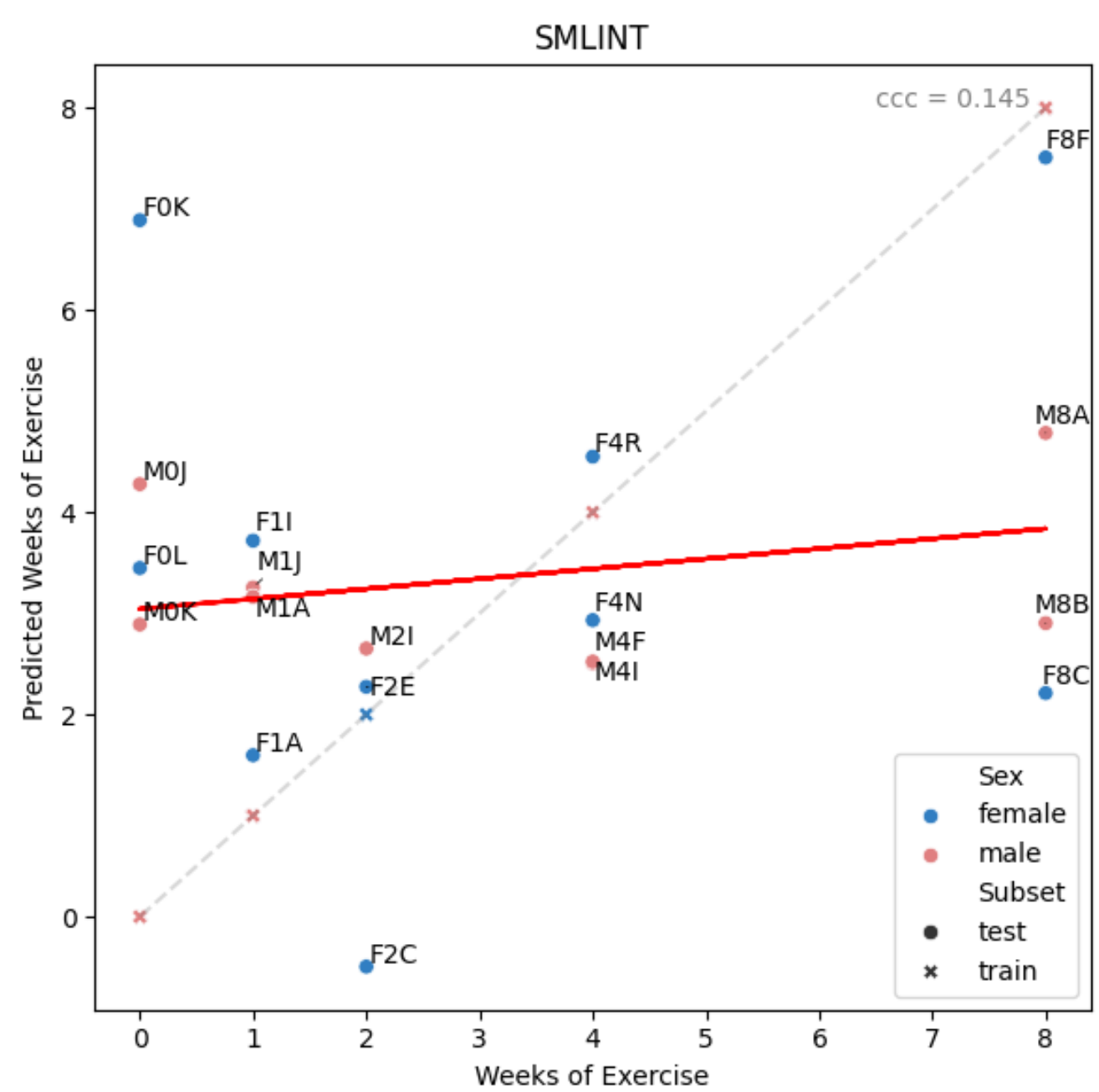

Fig. 22. Linear regression to predict weeks of exercise using RNA data from small intestine (SMLINT) alone. Points are colored by sex and shaped by train/test subset.

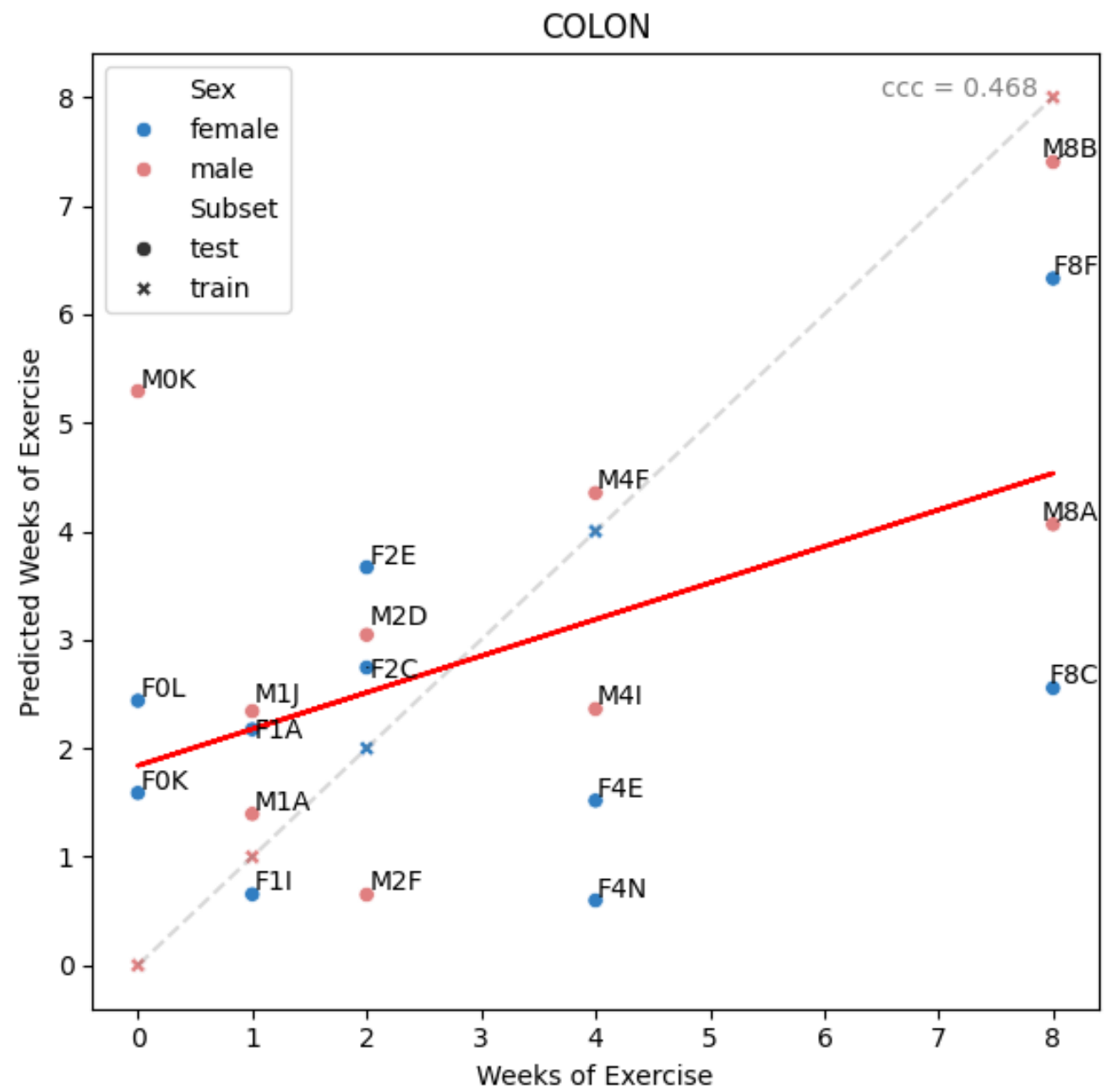

Fig. 23. Linear regression to predict weeks of exercise using RNA data from colon (COLON) alone. Points are colored by sex and shaped by train/test subset.

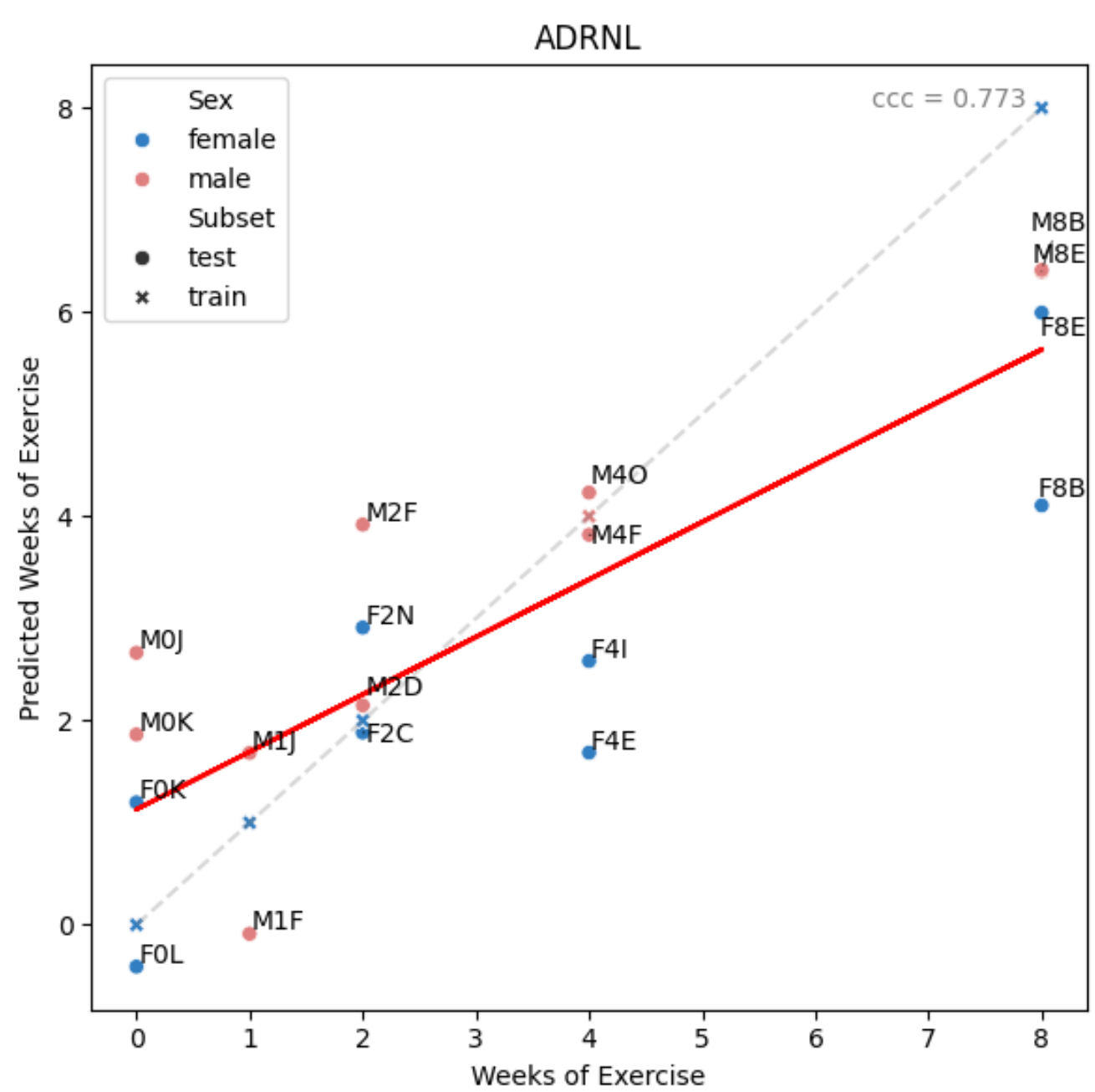

Fig. 24. Linear regression to predict weeks of exercise using RNA data from adrenal gland (ADRNL) alone. Points are colored by sex and shaped by train/test subset.

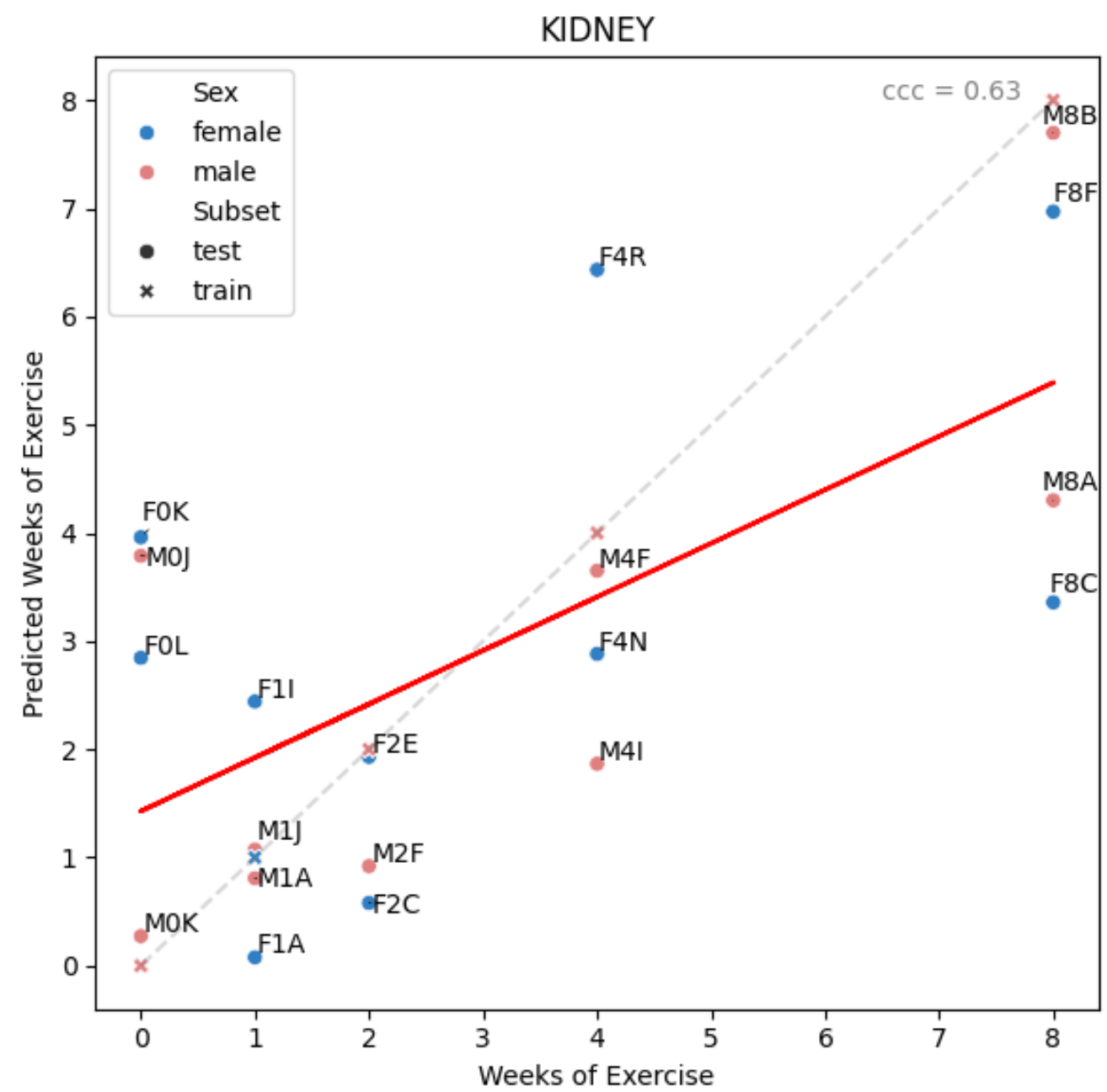

Fig. 25. Linear regression to predict weeks of exercise using RNA data from kidney (KIDNEY) alone. Points are colored by sex and shaped by train/test subset.

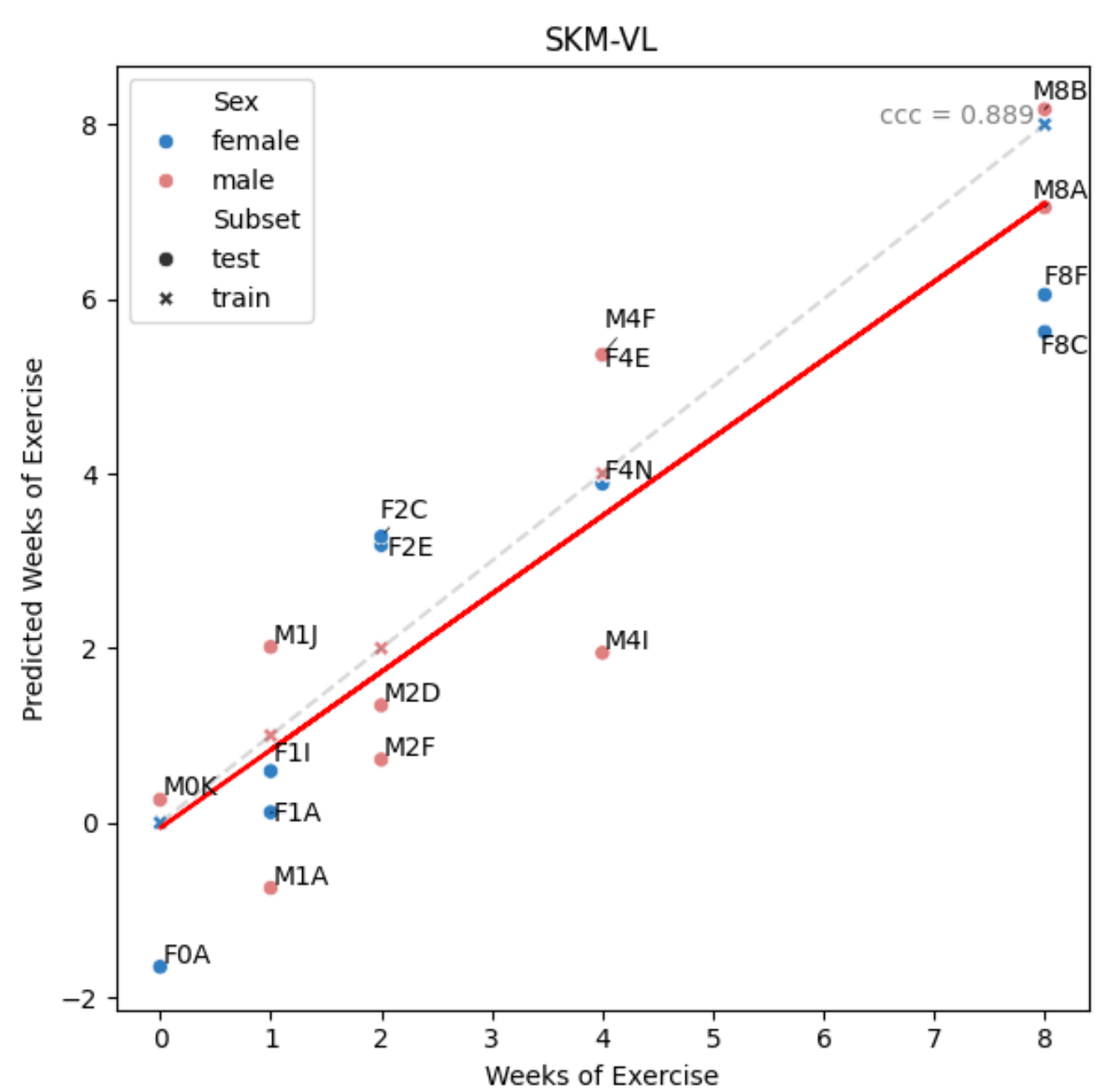

Fig. 26. Linear regression to predict weeks of exercise using RNA data from skeletal muscle vastus lateralis (SKM-VL) alone. Points are colored by sex and shaped by train/test subset.

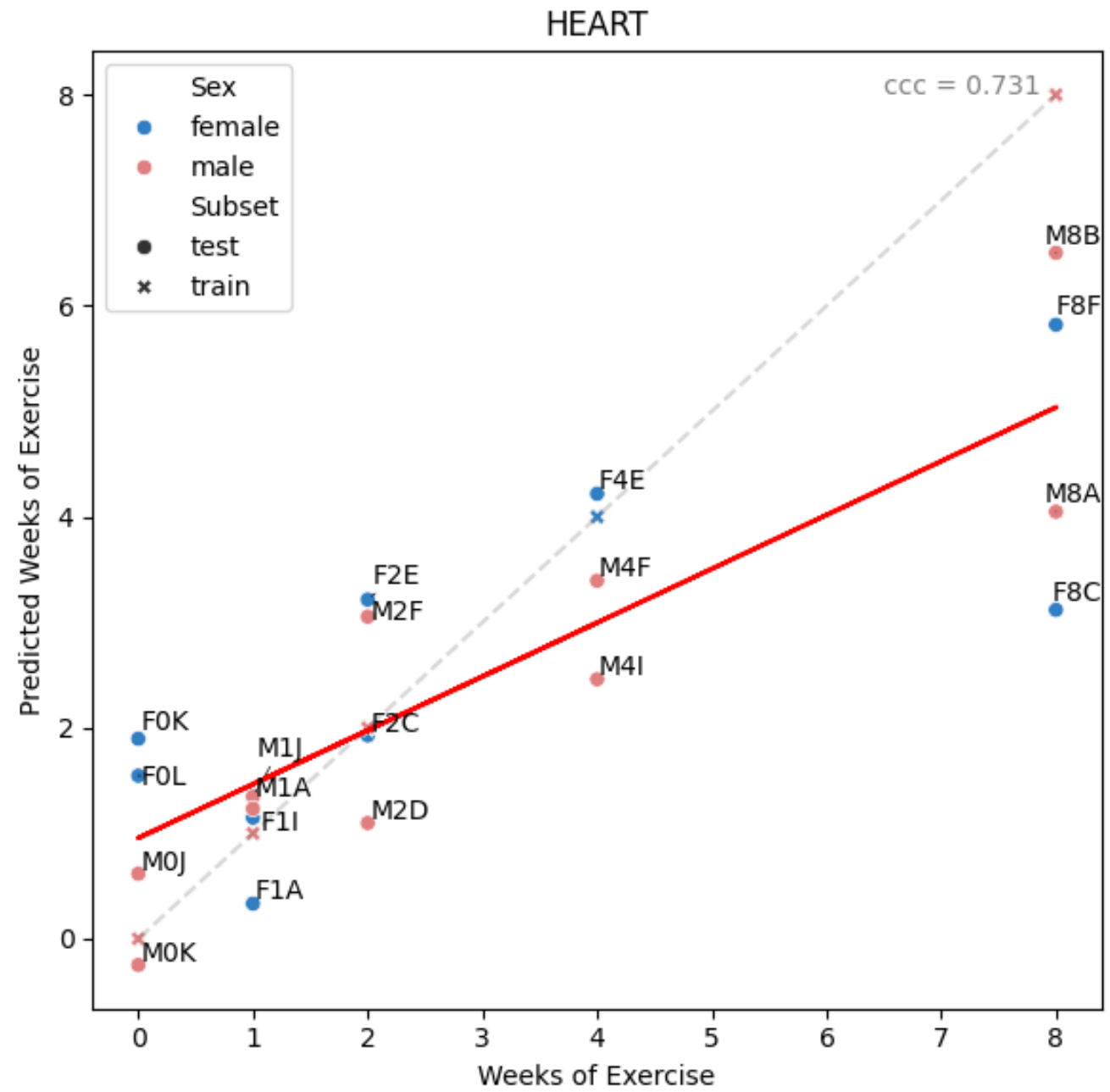

Fig. 27. Linear regression to predict weeks of exercise using RNA data from heart (HEART) alone. Points are colored by sex and shaped by train/test subset.

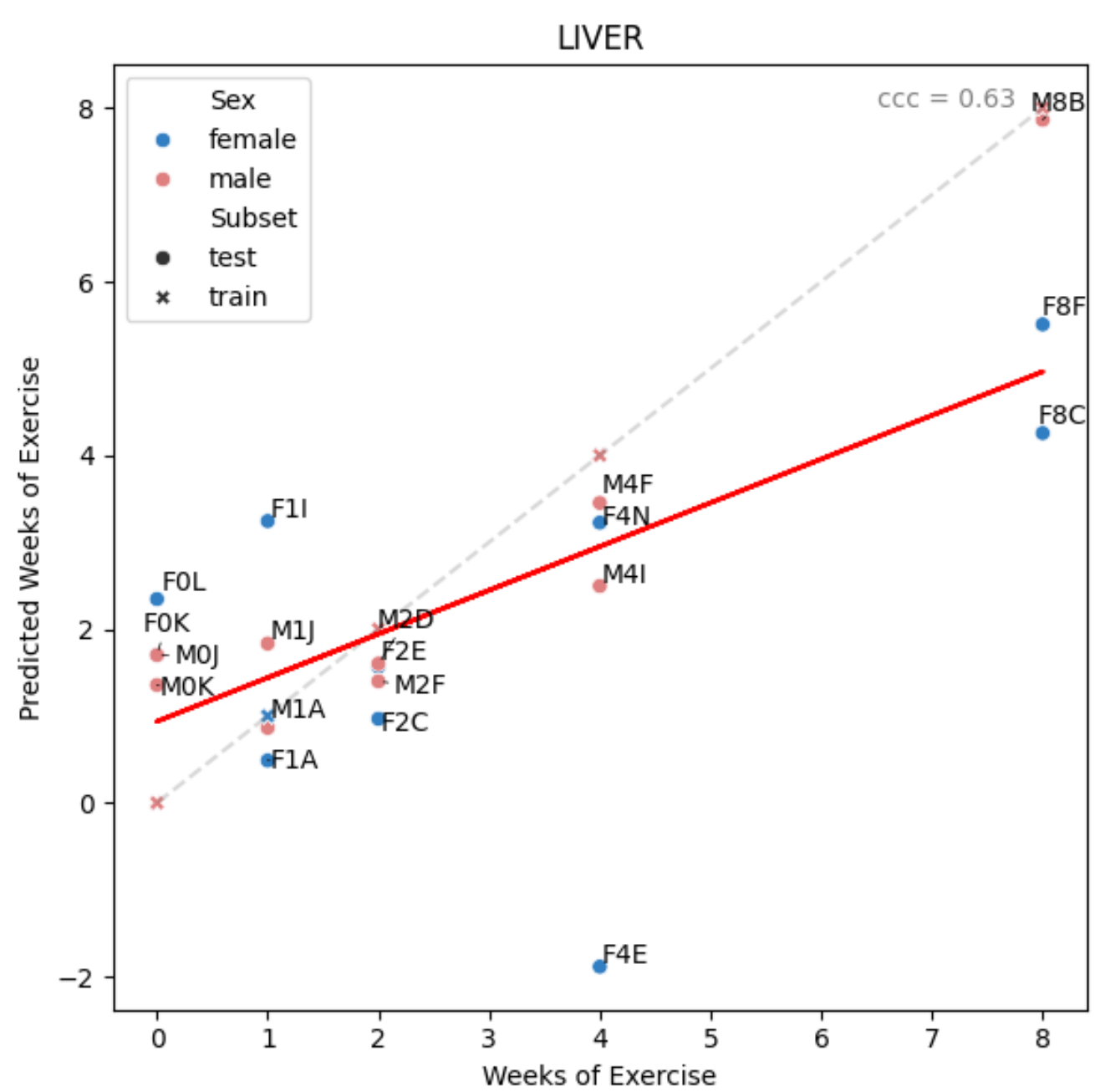

Fig. 28. Linear regression to predict weeks of exercise using RNA data from liver (LIVER) alone. Points are colored by sex and shaped by train/test subset.

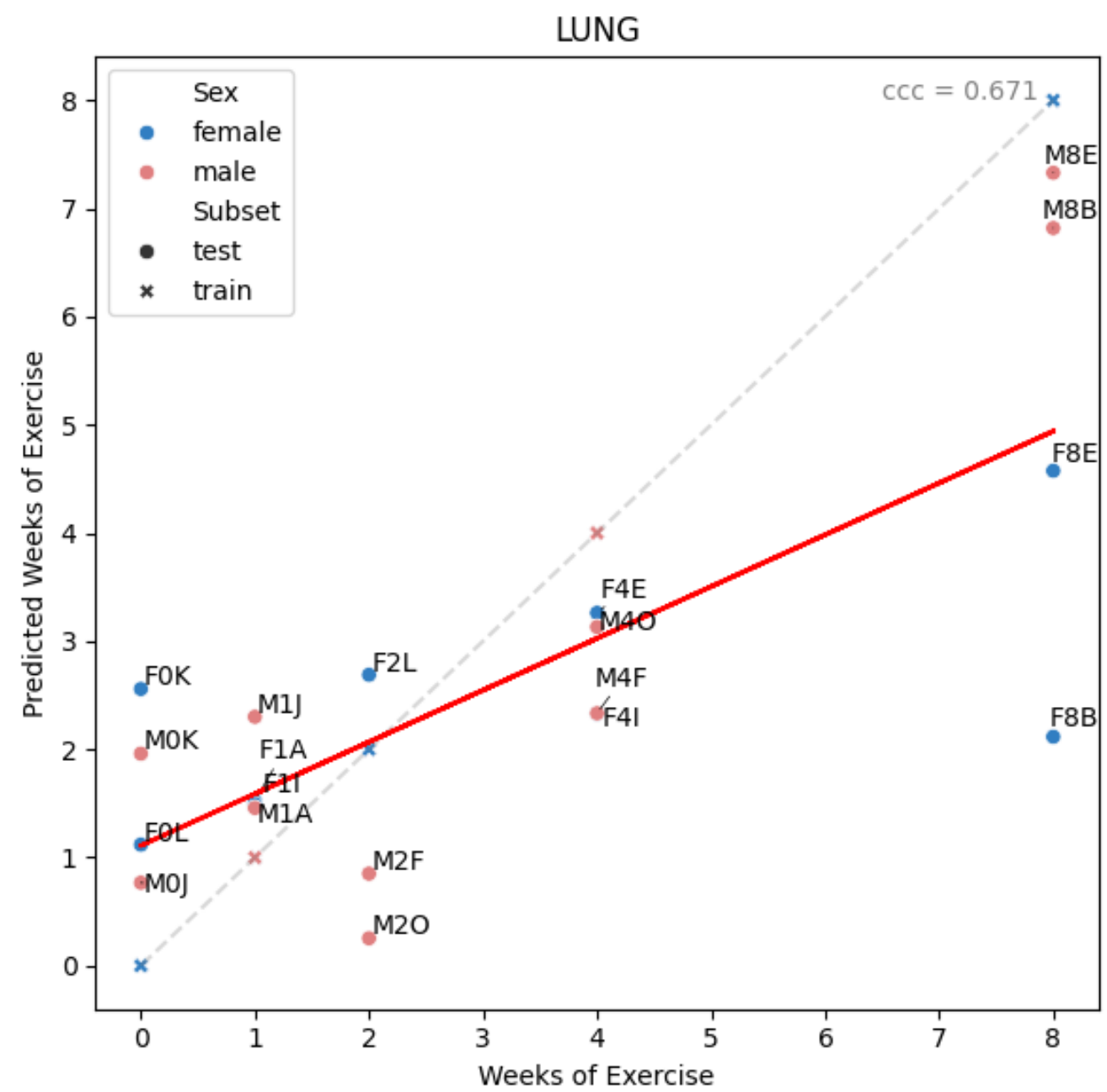

Fig. 29. Linear regression to predict weeks of exercise using RNA data from lung (LUNG) alone. Points are colored by sex and shaped by train/test subset.

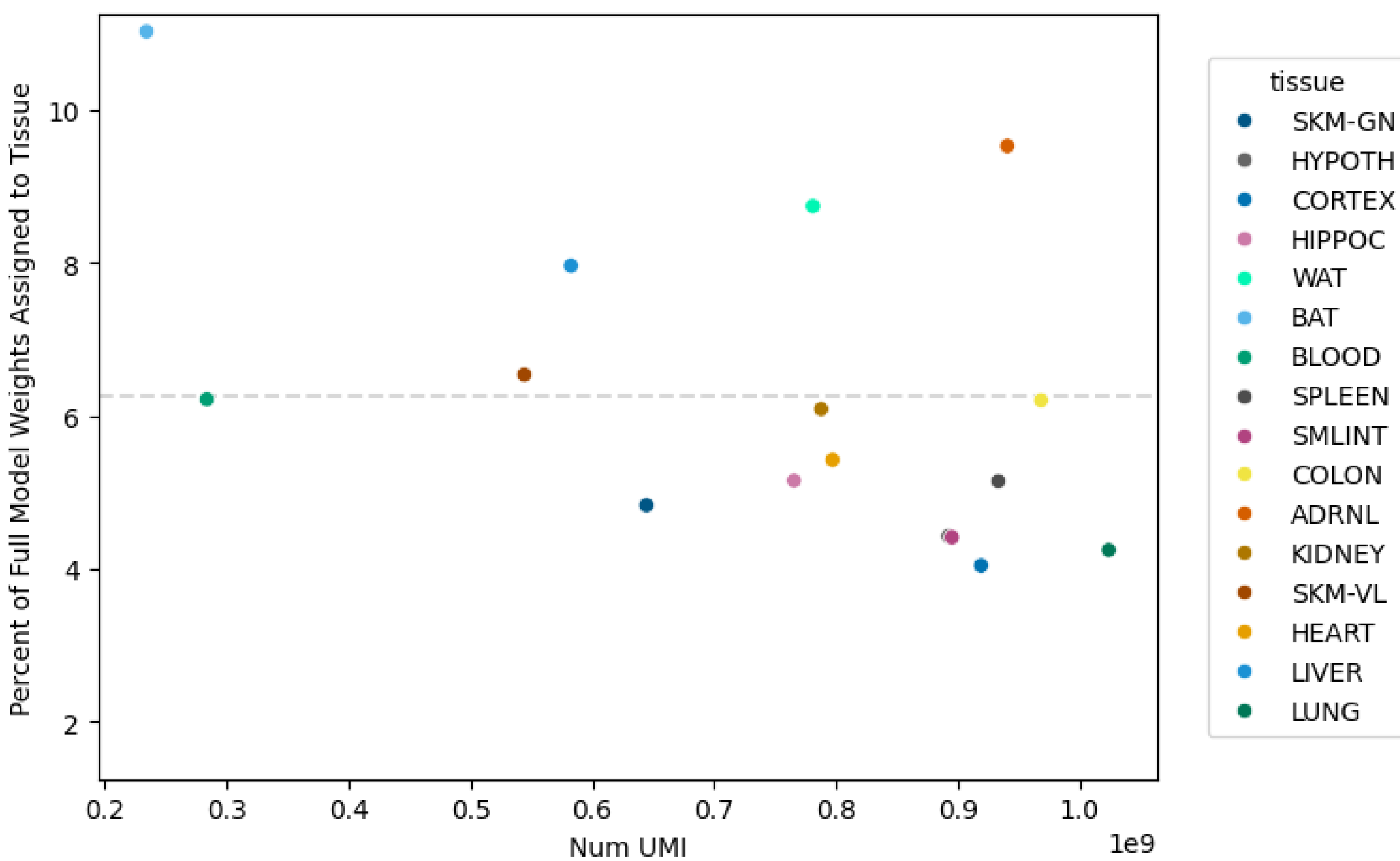

Fig. 30. Relationship between total UMI count per tissue and the percentage of full model weights assigned to that tissue in the multi-tissue RNA linear regression. The dotted line indicates the weight expected if each tissue contributed equally.

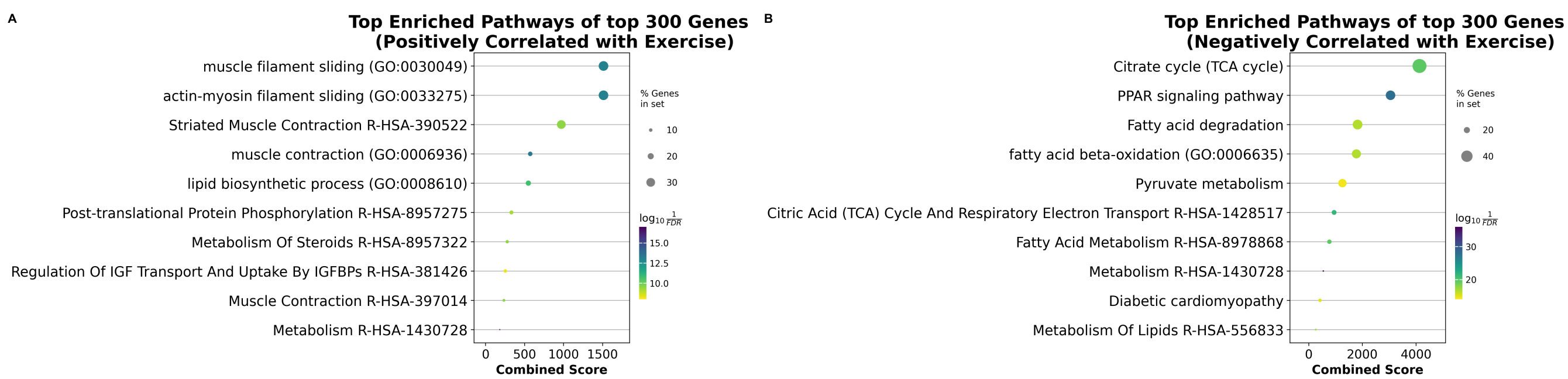

Fig. 31. Pathway enrichment analysis of genes with the highest absolute model weights in the RNA linear regression. A) Top enriched pathways among the top 300 genes positively correlated with weeks of exercise. B) Top enriched pathways among the top 300 genes negatively correlated with weeks of exercise.

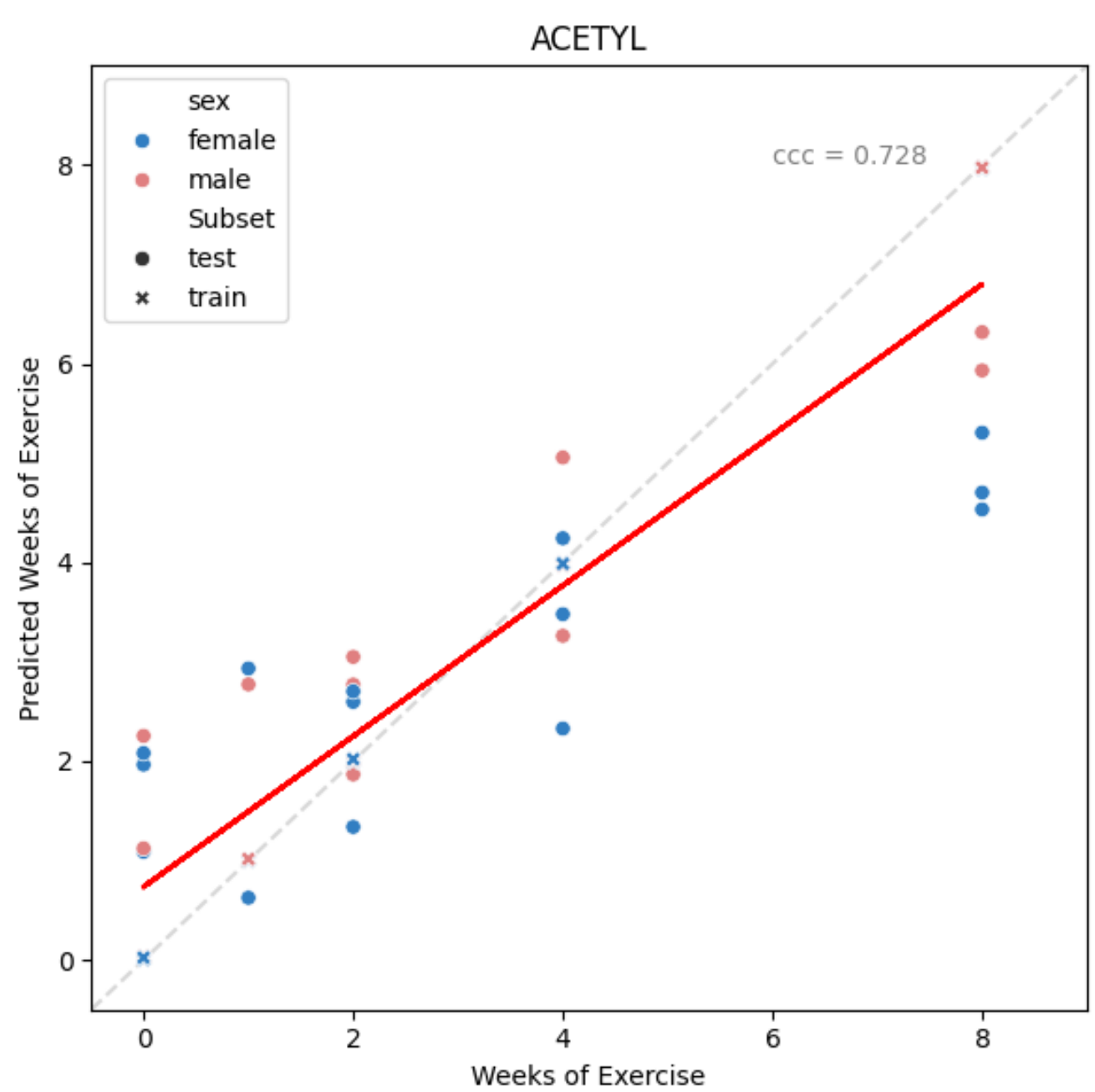

Fig. 32. Linear regression to predict weeks of exercise using acetylation (ACETYL) data alone. Predicted versus actual weeks of exercise, colored by sex and shaped by train/test subset.

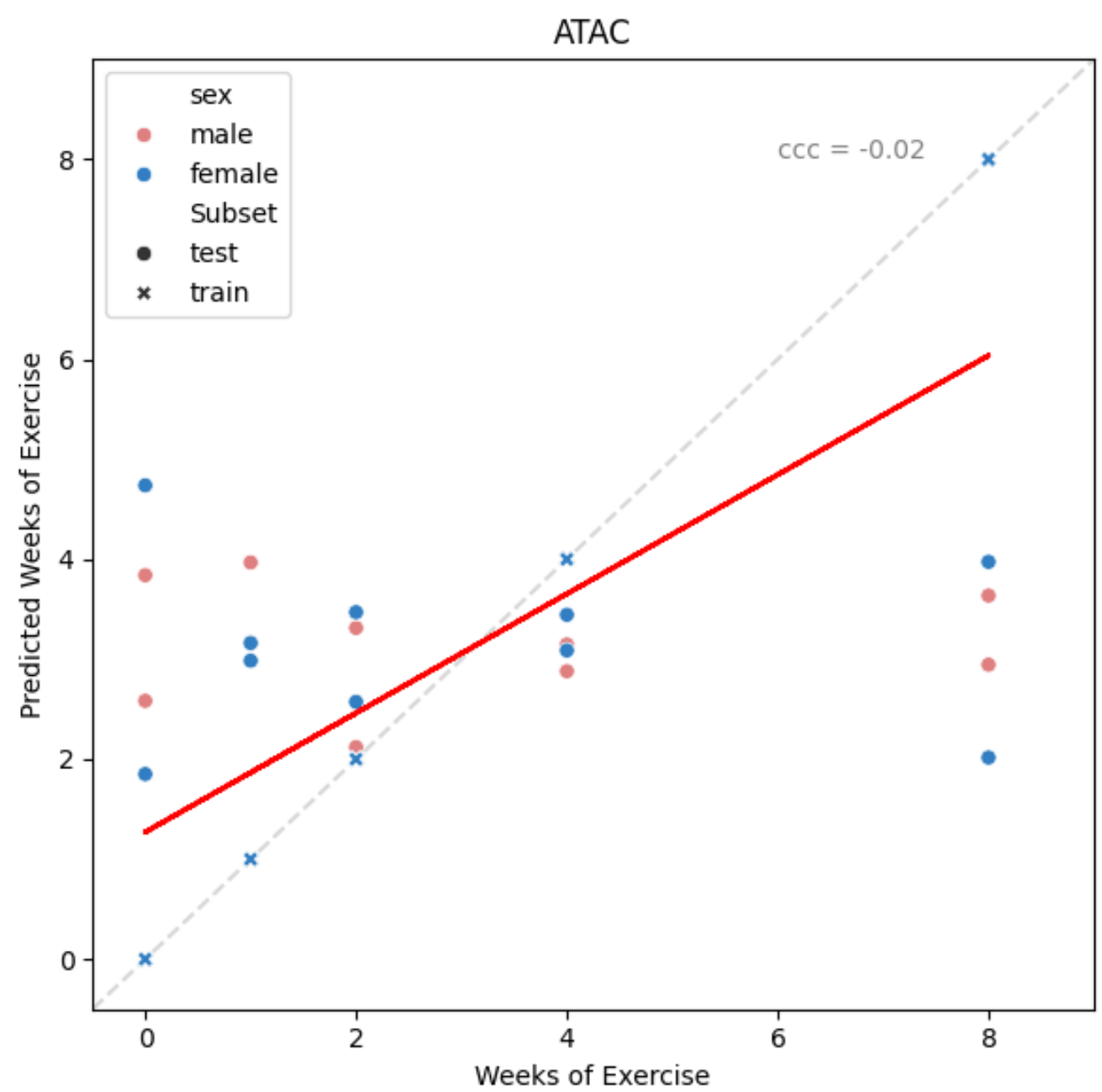

Fig. 33. Linear regression to predict weeks of exercise using ATAC-seq (chromatin accessibility) data alone. Predicted versus actual weeks of exercise, colored by sex and shaped by train/test subset.

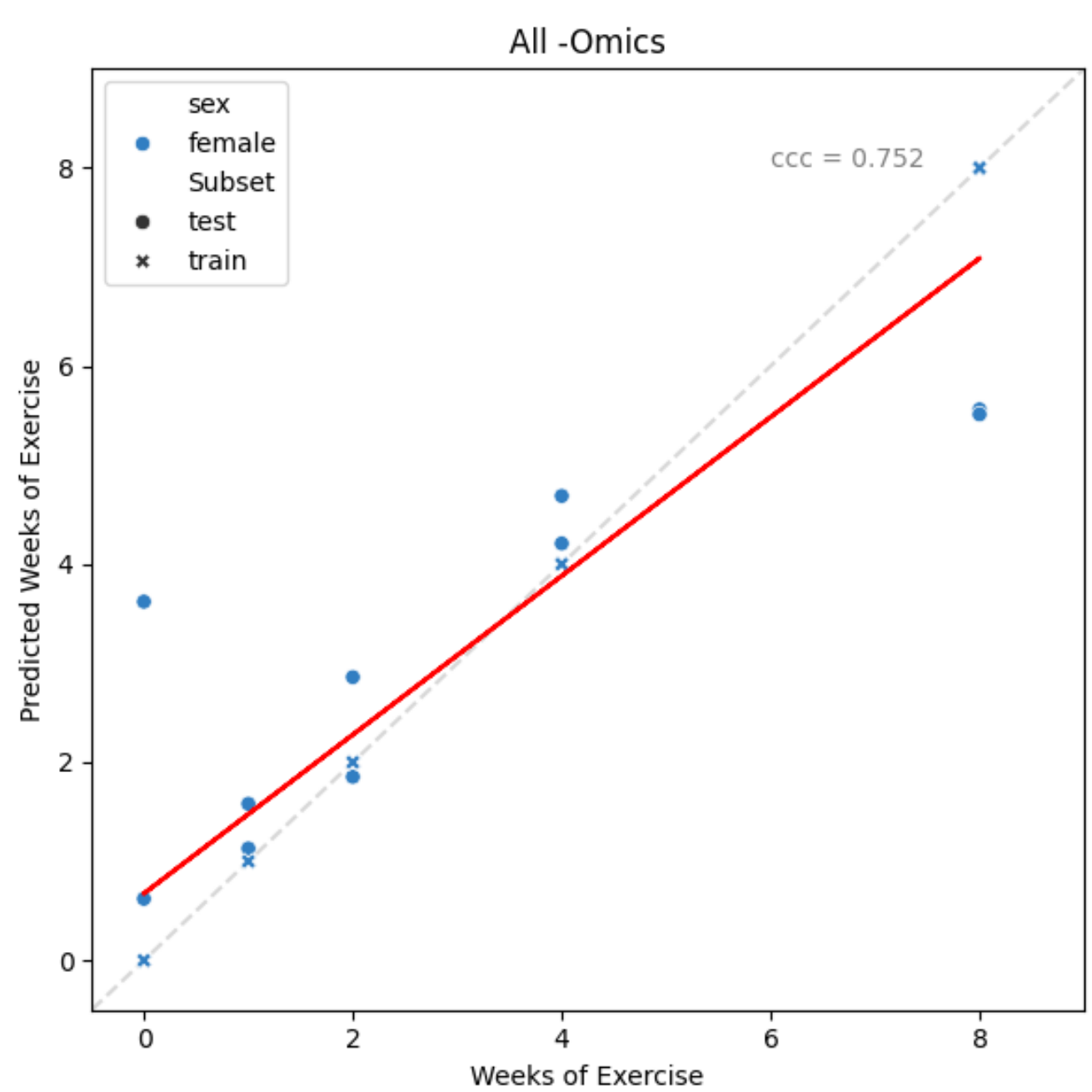

Fig. 34. Linear regression to predict weeks of exercise using all non-RNAseq omics combined. Used only Female Heart tissue due to lack of data from certain omics in other tissues/individuals. Predicted versus actual weeks of exercise, colored by sex and shaped by train/test subset.

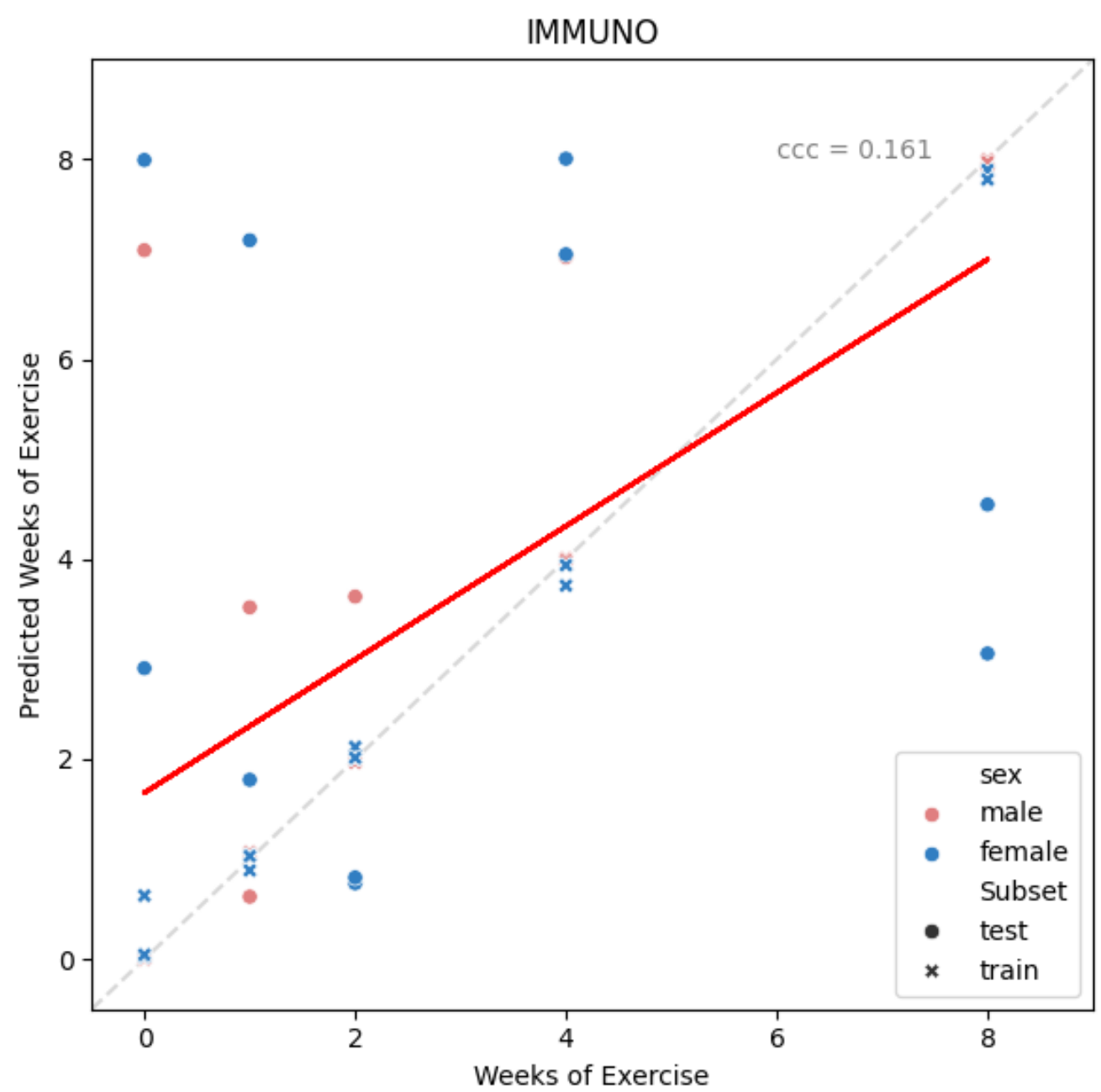

Fig. 35. Linear regression to predict weeks of exercise using immunoassay (IMMUNO) data alone. Predicted versus actual weeks of exercise, colored by sex and shaped by train/test subset.

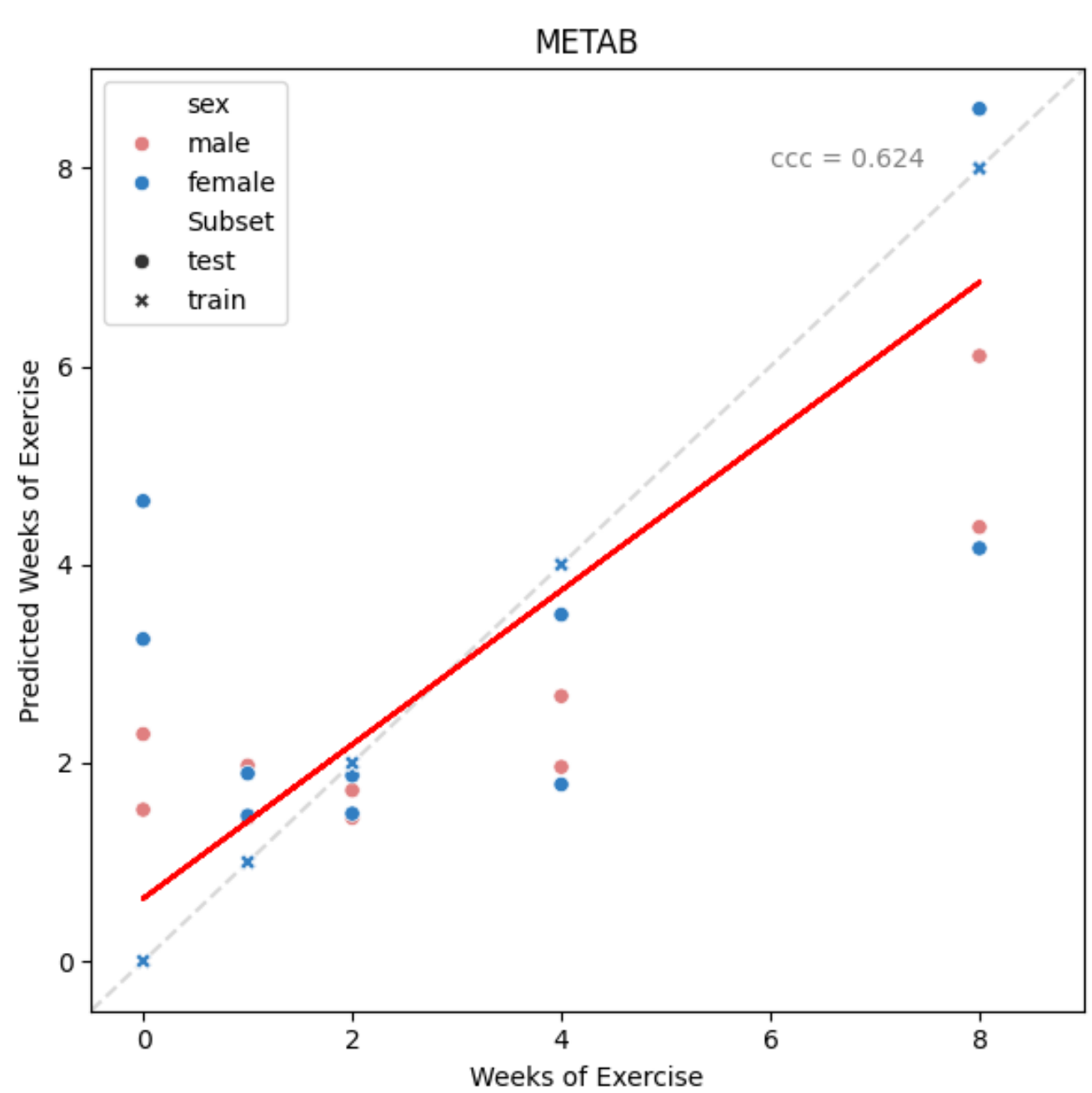

Fig. 36. Linear regression to predict weeks of exercise using metabolomics (METAB) data alone. Predicted versus actual weeks of exercise, colored by sex and shaped by train/test subset.

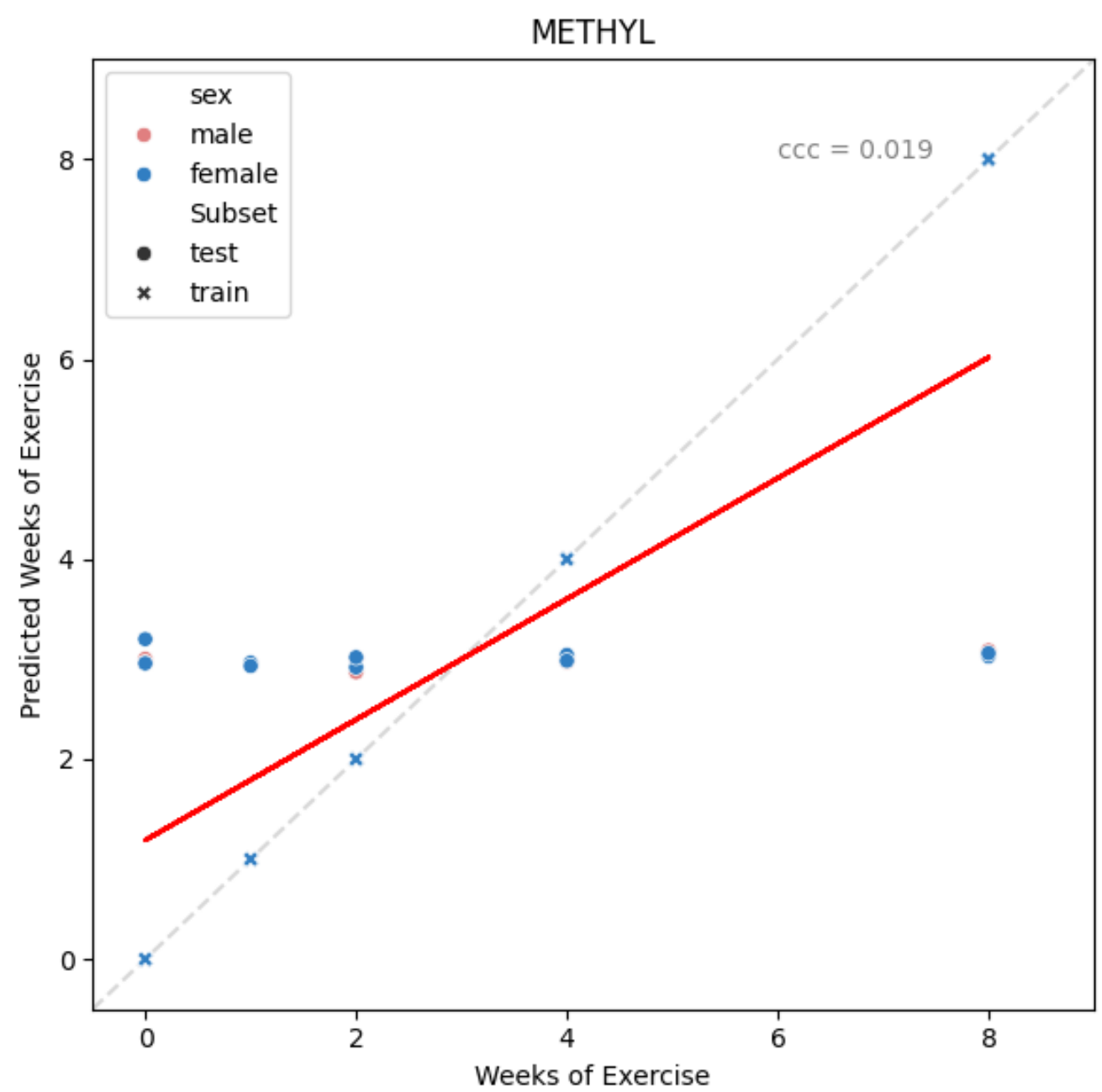

Fig. 37. Linear regression to predict weeks of exercise using DNA methylation (METHYL) data alone. Predicted versus actual weeks of exercise, colored by sex and shaped by train/test subset.

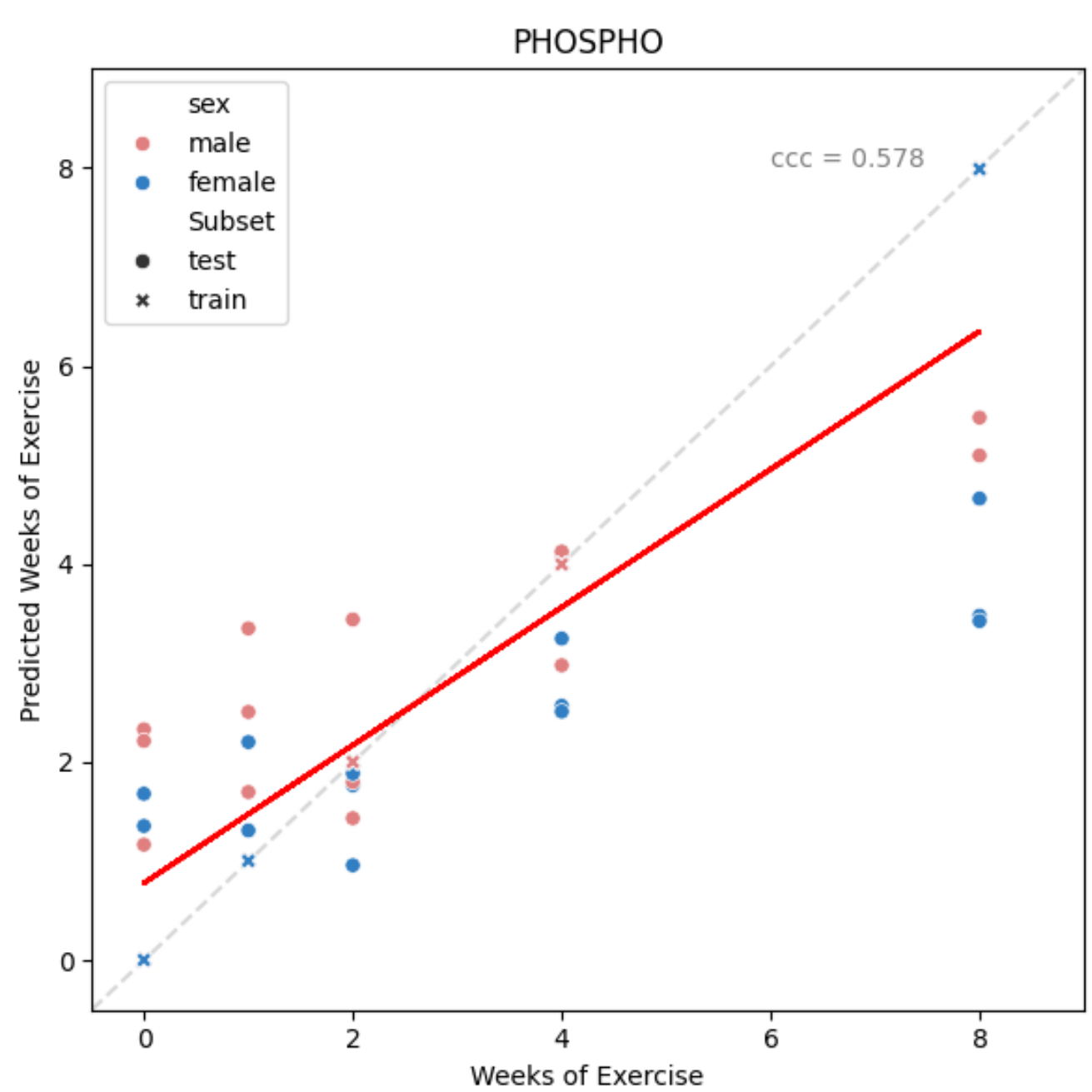

Fig. 38. Linear regression to predict weeks of exercise using phosphoproteomics (PHOSPHO) data alone. Predicted versus actual weeks of exercise, colored by sex and shaped by train/test subset.

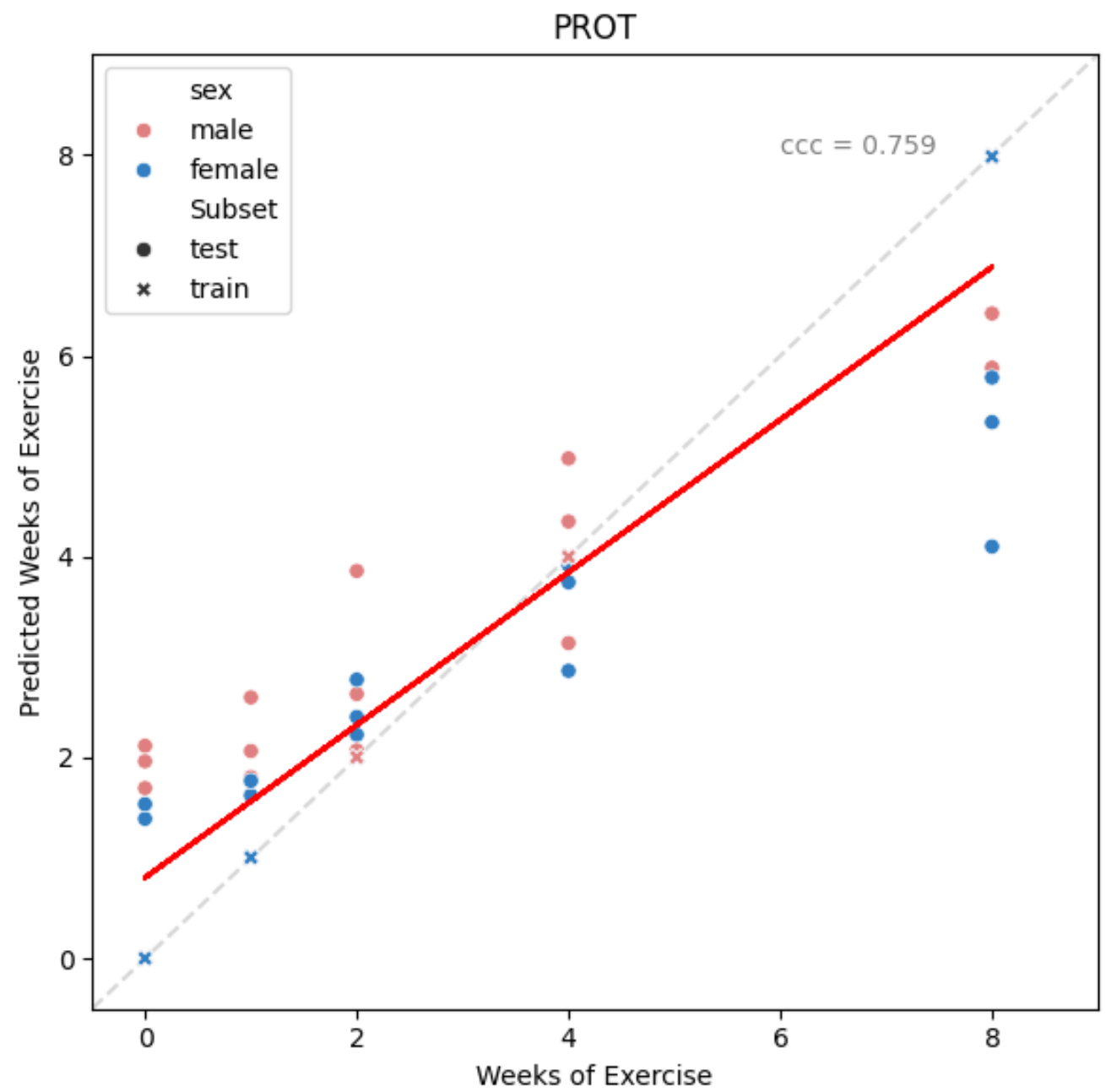

Fig. 39. Linear regression to predict weeks of exercise using proteomics (PROT) data alone. Predicted versus actual weeks of exercise, colored by sex and shaped by train/test subset.

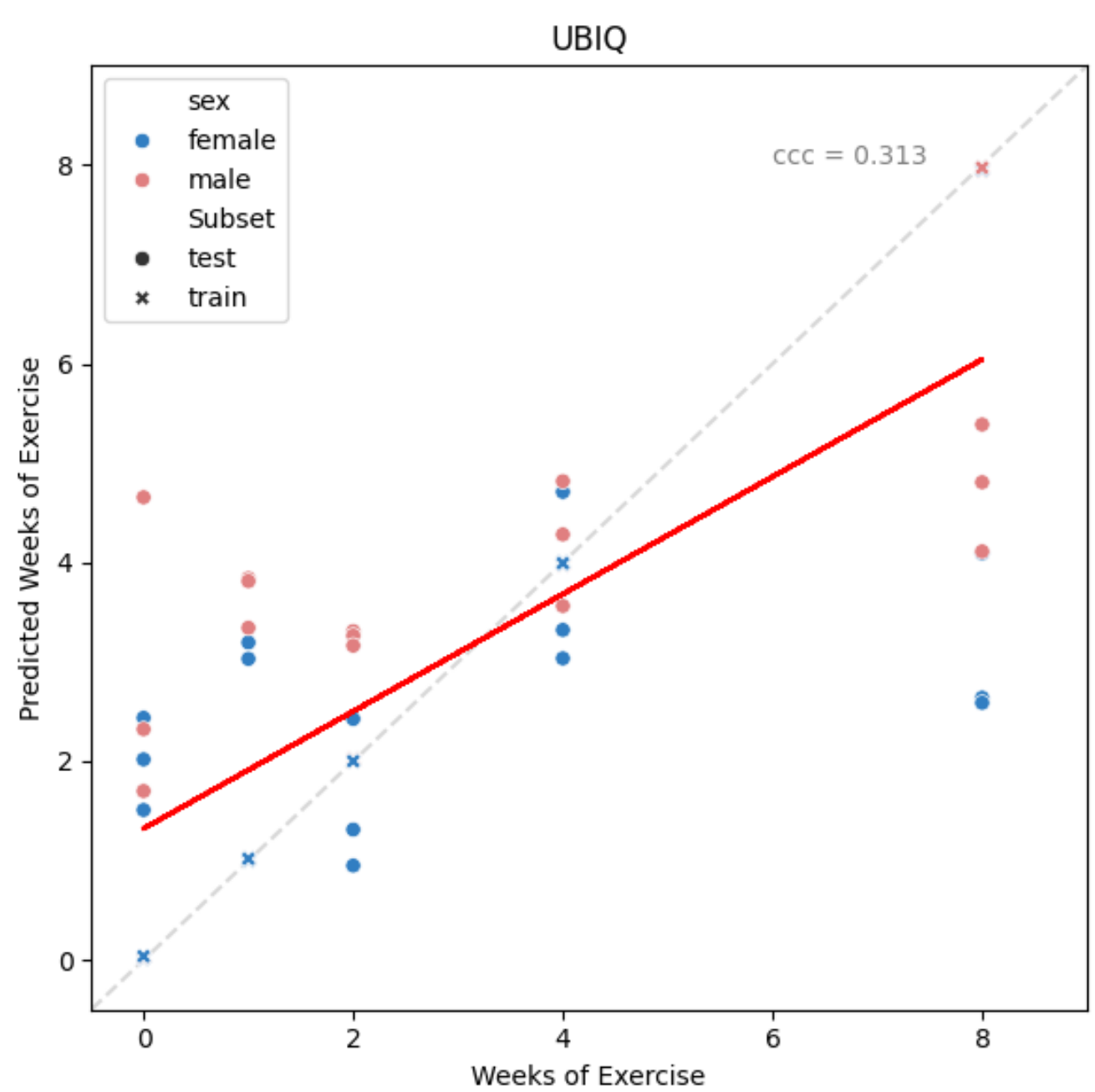

Fig. 40. Linear regression to predict weeks of exercise using ubiquitylation (UBIQ) data alone. Predicted versus actual weeks of exercise, colored by sex and shaped by train/test subset.
